# Supplementary material for: Aβ oligomer concentration in mouse and human brain and its drug-induced reduction ex vivo
Source: Cell Rep Med. 2022 May 17;3(5):100630. doi: 10.1016/j.xcrm.2022.100630 (PMC9133466; doi:10.1016/j.xcrm.2022.100630)
Supplement: Document S1. Figures S1–S6 and Table S1 [file mmc1.pdf]

Cell Reports Medicine, Volume *3*

Supplemental information

Aβ oligomer concentration in mouse and human brain and its drug-induced reduction *ex vivo*

Bettina Kass, Sarah Schemmert, Christian Zafiu, Marlene Pils, Oliver Bannach, Janine Kutzsche, Tuyen Bujnicki, and Dieter Willbold

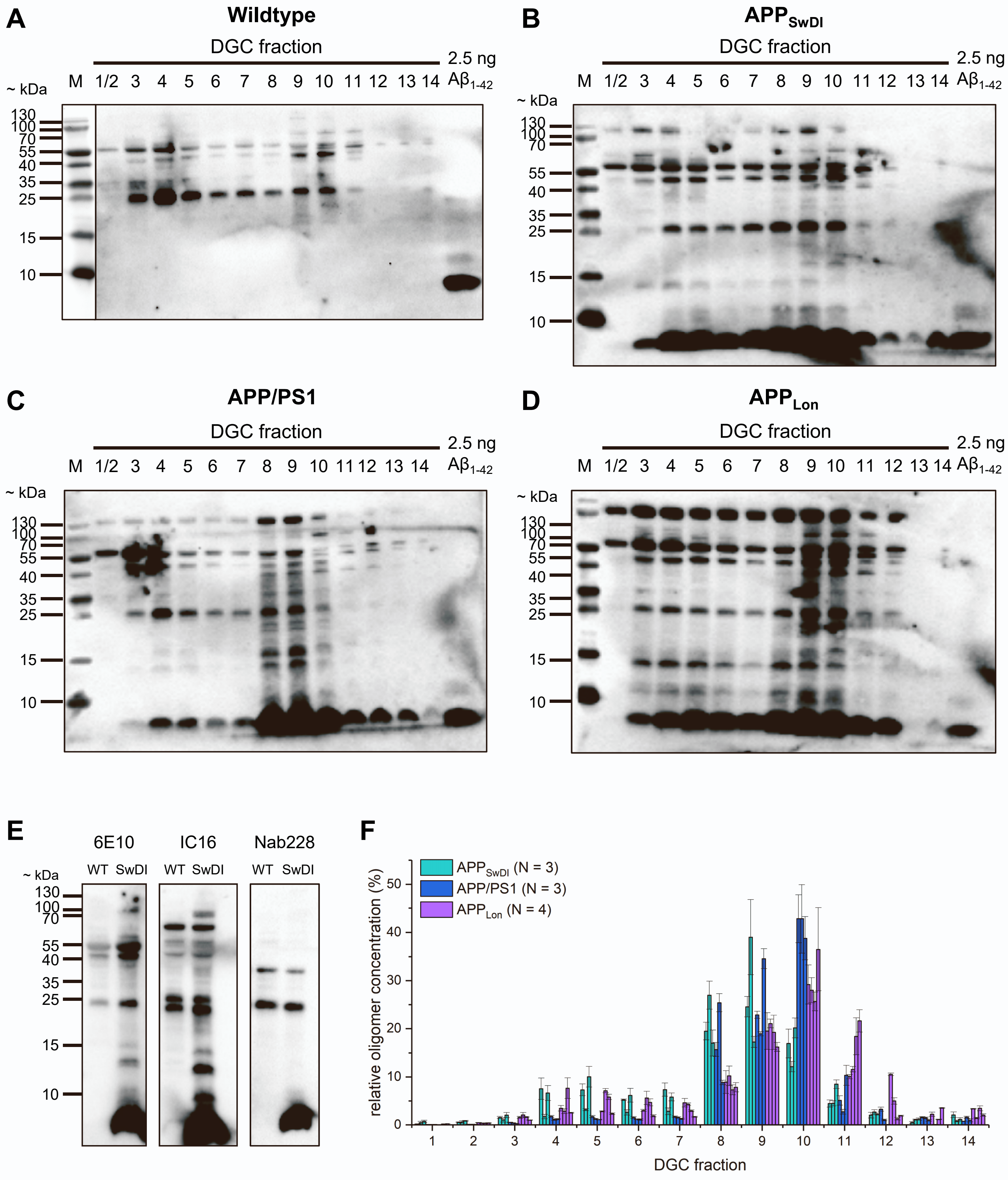

Figure S1: Full Western blots of mouse samples and sFIDA data from all individual animals, related to **Fig 1.**

(A) to (D): Western Blot analysis of undiluted DGC fractions (12 µl per lane) of one representative animal per group in comparison to a synthetic $A\beta_{1-42}$ standard. Blots were probed with monoclonal antibody 6E10. (A) wildtype, (B) APP_{SwDI} hom., (C) APP/PS1 het., (D) APP_{Lon} het.

(E): Brain homogenates of one wildtype or APP_{SwDI} mouse (30 µg protein per lane) were probed with monoclonal antibodies 6E10, IC16 or Nab228, each at a concentration of 1 µg/ml. (F): Relative Aβ oligomer concentrations of DGC fractions of different mouse models, displaying individual data points for each individual animal.

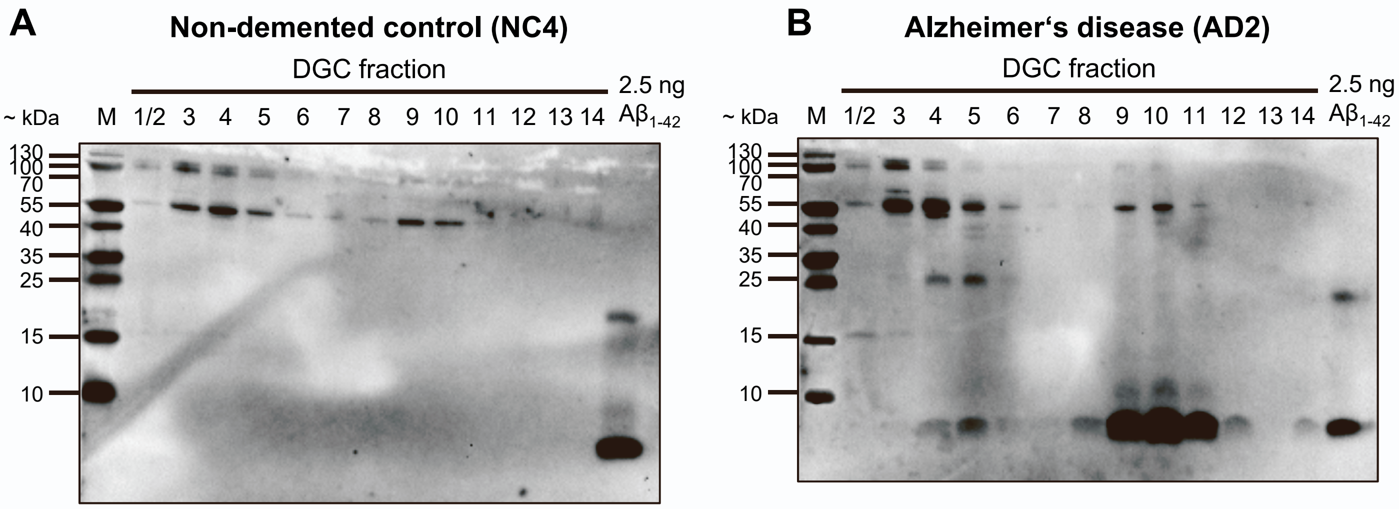

Figure S2: Full Western blots of human samples, related to **Fig 2.**
Western Blot analysis of undiluted DGC fractions (12 µl per lane) of one representative human brain sample per group in comparison to a synthetic $A\beta_{1-42}$ standard. (A) Non-demented control, (B) Alzheimer's disease. Blots were probed with monoclonal antibody 6E10 at a concentration of 1 µg/ml.

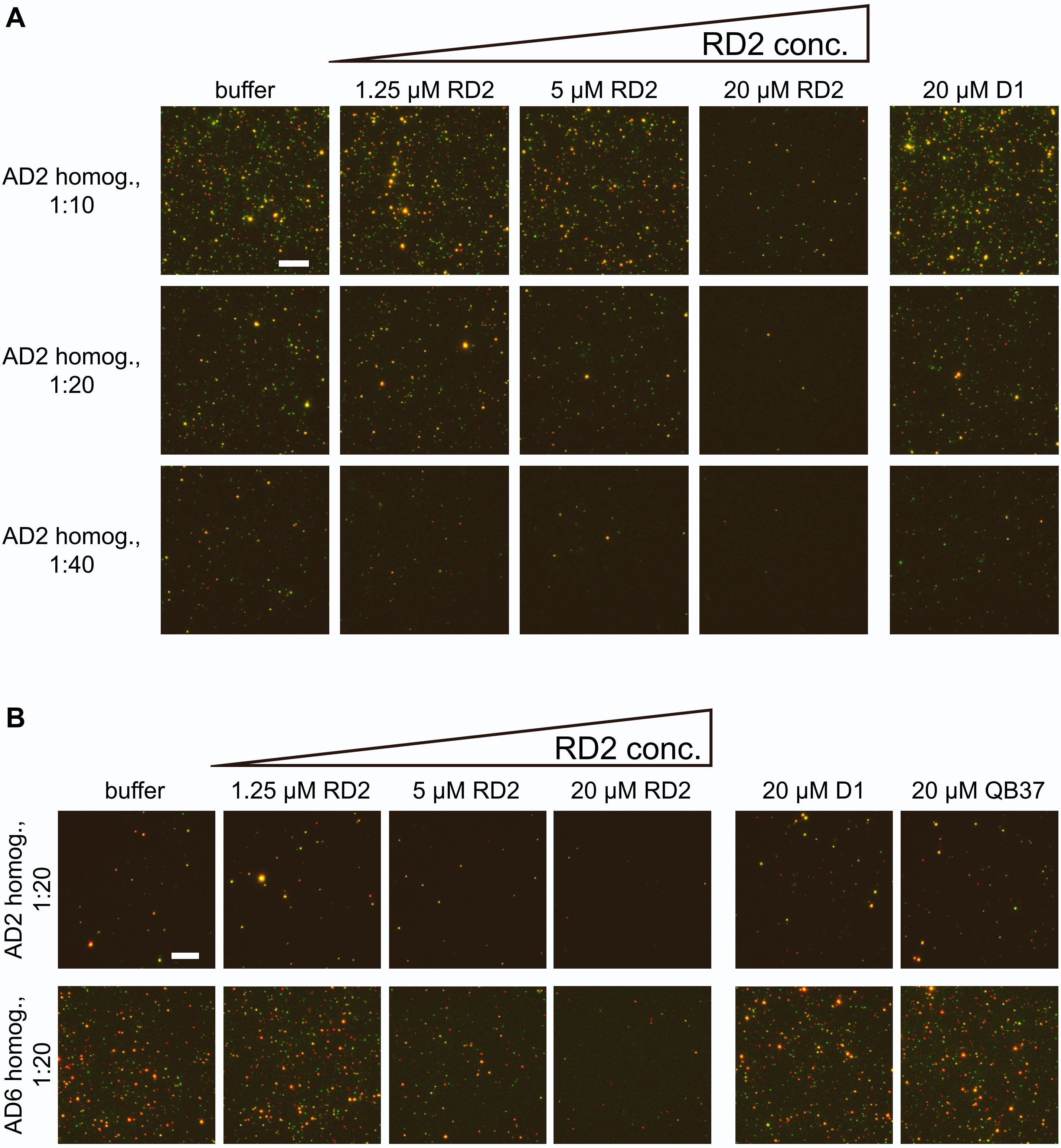

Figure S3: Representative TIRF image sections of human brain homogenate after one day of incubation with D-peptides, related to **Fig 4** and **Fig 5**. Panel (A), corresponding to Fig 4: Homogenates were diluted 1:10, 1:20 or 1:40 during incubation with D-peptides. Final dilution factors during image acquisition were 1:20, 1:40, and 1:80, respectively. Red and green fluorescence channels were merged. The scale bar represents 10 µm. Panel (B): Representative TIRF image sections of human brain homogenate after one day of incubation with D-peptides, corresponding to the data presented in **Fig 5**. Homogenates were diluted 1:20 during incubation with D-peptides. The final dilution factor during image acquisition was 1:40. Red and green fluorescence channels were merged. The scale bar represents 10 µm.

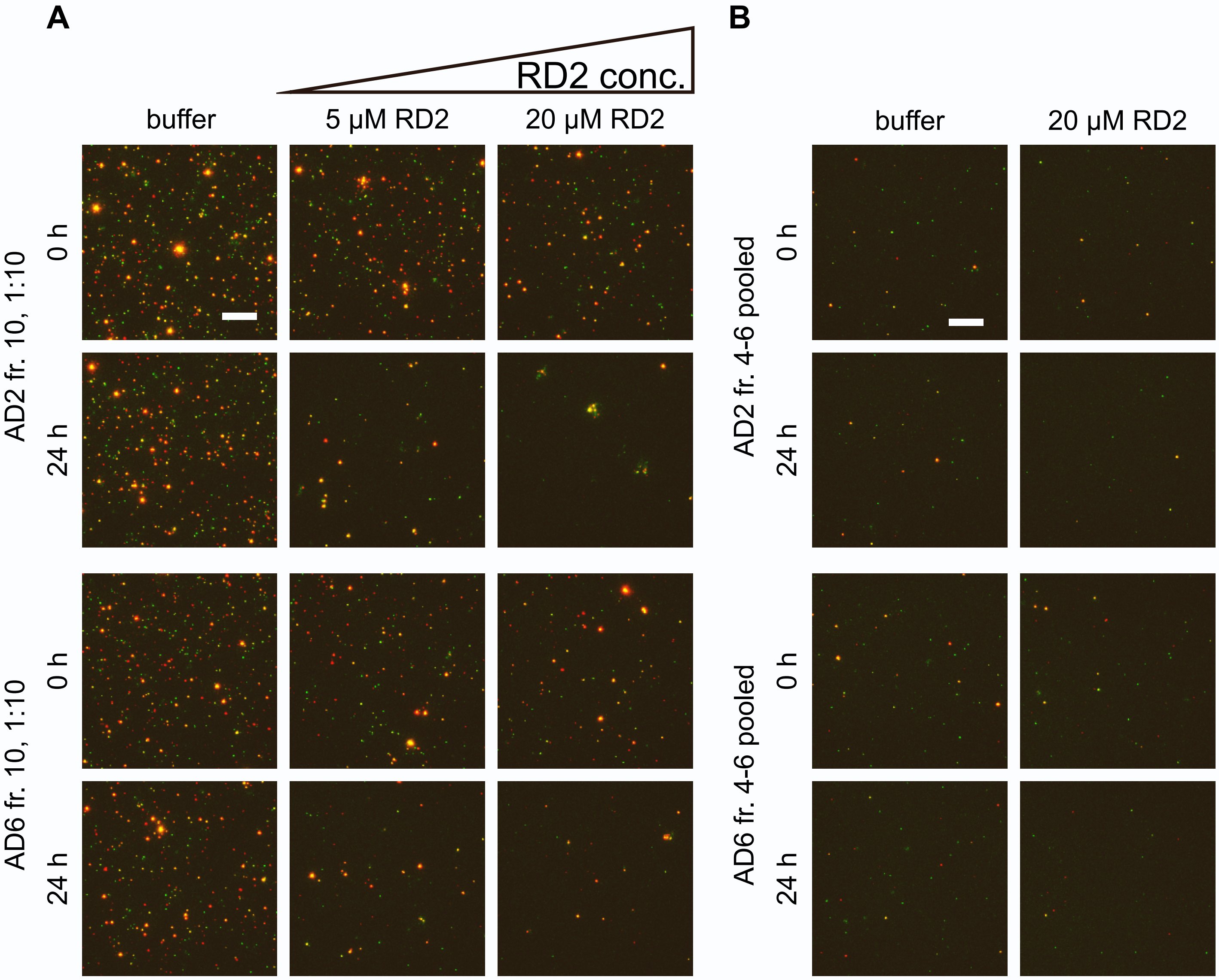

Figure S4, related to **Fig 6:** Representative TIRF image sections of DGC-obtained fractions of human brain homogenate after one day of incubation with RD2. Red and green fluorescence channels were merged. The scale bar represents 10 µm. (A) Images corresponding to the analysis of ex vivo target engagement in fraction 10 of human AD brain homogenates. Fraction 10 was diluted 1:10 during incubation with D-peptides. The final dilution factor during image acquisition was 1:20 (B) Images corresponding to the analysis of ex vivo target engagement in pooled fractions 4 to 6 of human AD brain homogenates. The final dilution factor during image acquisition was 1:2.

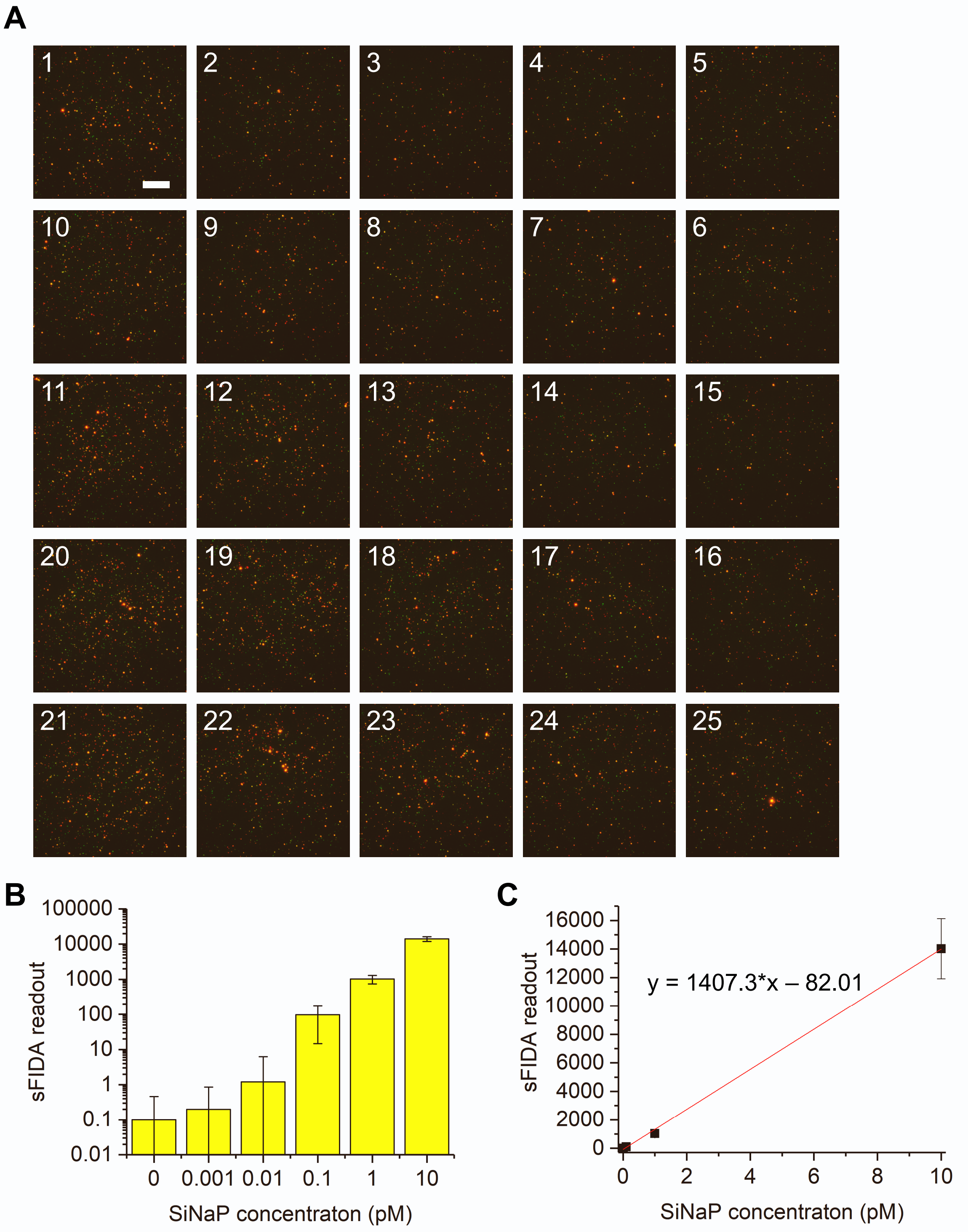

D

$$LOD[fM] = \frac{(sFIDA\,readout_{0\,pM} + 3 \times std\,dev_{0\,pM}) - b}{m} \times 1000$$

$$LLOQ[fM] = \frac{(sFIDA\,readout_{0\,pM} + 10 \times std\,dev_{0\,pM}) - b}{m} \times 1000$$

Figure S5, related to **STAR methods:** Acquisition of sFIDA image data and calculation of concentrations using silica nanoparticle (SiNaP) standards. (A) 25 TIRF images per well are taken in the red and green channel in the order indicated by the numbers 1 to 25. Each sample is measured in triplicate, resulting in a total of 75 images used for analysis. Overlays of red and green channels are shown. The sample depicted here is human AD brain homogenate, diluted 1:20 – corresponding to a final dilution during image acquisition of 1:40. The scale bar represents 20 µm. (B) Co-localized pixels exceeding the background intensity threshold based on the 0.01% pixels with the highest intensity in the buffer control (0 pM SiNaP) are counted to calculate the sFIDA readout. Results in (B) and (C) are displayed as mean ± SD, N = 3 (technical replicates). Please note the logarithmic scale in (B). (C) A serial dilution of SiNaPs coated with a defined number of A$\beta_{1\text{-}15}$ epitopes is used to calculate the concentration of biological samples by linear regression. (D) The limit of detection (LOD) and lower limit of quantitation (LLOQ) are calculated using the slope *m* and Y-axis intercept *b* of the linear regression line.

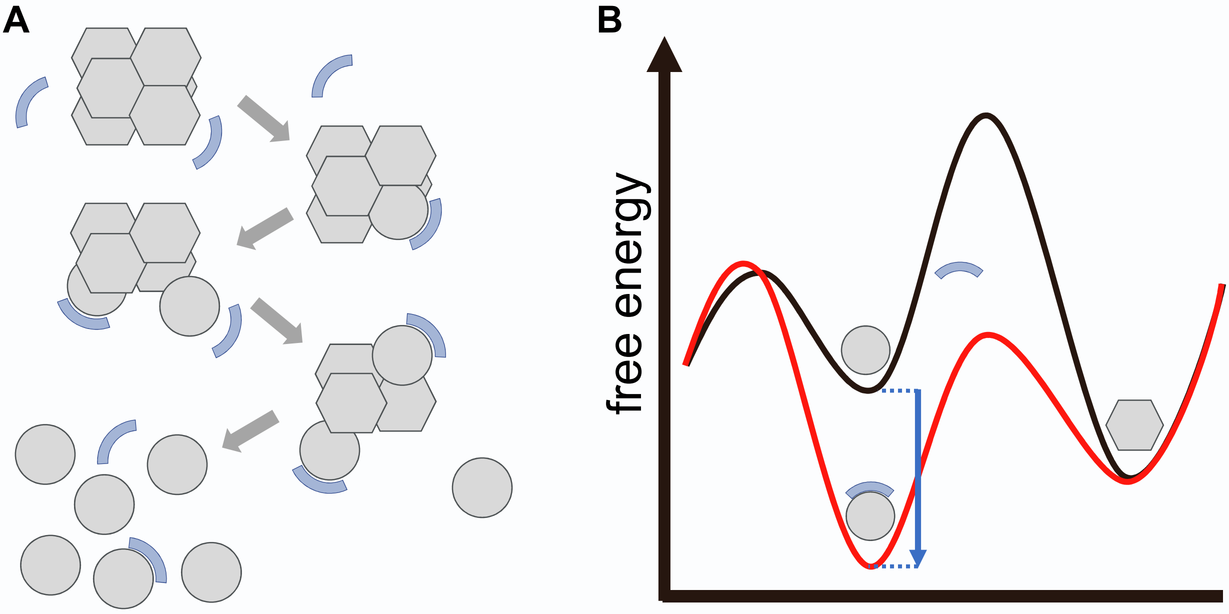

Figure S6, related to **Fig 5:** Proposed mode of action for RD2. (A) RD2 was designed to stabilize Aβ monomers in their native, intrinsically disordered conformation – symbolized by circles. This conformation is distinct from the yet unknown, but certainly highly defined beta-sheet-rich conformation of Aβ building blocks in Aβ oligomers[1] – symbolized by hexagons. Explanation from top to bottom: RD2 molecules – symbolized by circle segments – approach Aβ oligomers. Due to their affinity to Aβ monomers, each RD2 molecule will interact with one of the Aβ building blocks within the Aβ oligomer assembly and thereby pushes its conformation towards the intrinsically disordered monomer conformation. This is incompatible with the oligomer assembly and therefore destabilizing the oligomer assembly. Further destabilization by interaction of additional RD2 molecules with other monomer building blocks, ultimately leads to the complete disassembly of the oligomer into Aβ monomers in their native intrinsically disordered conformation. The affinity between RD2 and Aβ monomers is in the nanomolar KD range although both molecules remain disordered in this transient complex[2], which may therefore be called "fuzzy complex"[3]. The mode of action as shown above, predicts three important properties of RD2. First, because less RD2 molecules are needed than Aβ monomer building blocks are present, RD2 acts at sub-stoichiometric concentrations. We have shown previously, that even at 1:10 sub-stoichiometric ratios, RD2 is efficiently disassembling Aβ oligomers. Second, such destabilization of oligomers by RD2 can be expected to be cooperative. Indeed, we found a Hill coefficient of 3[2]. Third, because RD2 is practically folding Aβ building blocks from oligomers back to their monomeric conformation, RD2 is acting similar to chaperone. Whether this may be called a truly catalytic activity remains to be demonstrated. We called this mode of action "anti-prionic", because it is ultimately disrupting prion-like behaving aggregates[4]. (B) Qualitative and schematic free energy landscape for the anti-prionic mode of action. The black line represents the energy landscape in absence of RD2. Aβ oligomers are more stable than monomers. This allows the formation of oligomers from monomers thermodynamically, although there is a kinetic barrier, which is called primary nucleation and currently under intensive investigation[5,6]. Stabilization of the Aβ monomer by RD2 is lowering the free energy of the Aβ monomer (red line), when in complex with RD2 by the free binding energy (blue arrow) of the complex. Thus, in the presence of RD2, the monomer has a lower free energy as compared to the oligomer, oligomers are disassembled with a reaction rate that is RD2 and Aβ oligomer concentration dependent, exactly as is demonstrated here in this work (Figure 5).

1. König AS, Rösener NS, Gremer L, Tusche M, Flender D, Reinartz E, Hoyer W, Neudecker P, Willbold D, Heise H. Structural details of amyloid β oligomers in complex with human prion protein as revealed by solid-state MAS NMR spectroscopy. J Biol Chem. 2021;296:100499.
2. Zhang T, Gering I, Kutzsche J, Nagel-Steger L, Willbold D. Toward the mode of action of the clinical stage all-D-enantiomeric peptide RD2 on Aβ42 aggregation. ACS Chem Neurosci. 2019;10(12):4800-9.
3. Borgia A, Borgia MB, Bugge K, Kissling VM, Heidarsson PO, Fernandes CB, Sottini A, Soranno A, Buholzer KJ, Nettels D, et al. Extreme disorder in an ultrahigh-affinity protein complex. Nature. 2018;555(7694):61-6.
4. Willbold D, Kutzsche J. Do we need anti-prion compounds to treat Alzheimer's disease? Molecules. 2019;24(12).
5. Noji M, Samejima T, Yamaguchi K, So M, Yuzu K, Chatani E, Akazawa-Ogawa Y, Hagihara Y, Kawata Y, Ikenaka K, et al. Breakdown of supersaturation barrier links protein folding to amyloid formation. Communications Biology. 2021;4(1):120.
6. Meisl G, Kirkegaard JB, Arosio P, Michaels TCT, Vendruscolo M, Dobson CM, Linse S, Knowles TPJ. Molecular mechanisms of protein aggregation from global fitting of kinetic models. Nature Protocols. 2016;11(2):252-72.

Table S1: Summary of statistical analyses, related to **STAR methods, Fig. 3, Fig. 4, Fig. 5, Fig. 6**

Figure	ANOVA	*Post hoc* analysis	Degrees of freedom	
3B	Kruskal-Wallis One-way ANOVA on ranks	Student-Newman-Keuls	4	H: 12.233, P = 0.016
4A	One-way ANOVA	Student-Newman-Keuls	8	F: 7.513, P = <0.001
4B	One-way ANOVA	Student-Newman-Keuls	8	F: 15.667, P = <0.001
4C	One-way ANOVA	Student-Newman-Keuls	8	F: 30.803, P = <0.001
5A	Kruskal-Wallis One-way ANOVA on ranks	Student-Newman-Keuls	5	H: 13.865, P = 0.016
5C	Kruskal-Wallis One-way ANOVA on ranks	Student-Newman-Keuls	5	H: 15.339, P = 0.009
5E	Kruskal-Wallis One-way ANOVA on ranks	Student-Newman-Keuls	5	H: 12.977, P = 0.024
5G	Kruskal-Wallis One-way ANOVA on ranks	Student-Newman-Keuls	5	H: 15.129, P = 0.010
6A	Kruskal-Wallis One-way ANOVA on ranks	Student-Newman-Keuls	2	H: 7.200, P= 0.004
6B	Kruskal-Wallis One-way ANOVA on ranks	Student-Newman-Keuls	2	H: 7.200, P= 0.004
6C	-	t-test, two-tailed	4	t: 16.876, P = <0.001
6D	-	t-test, two-tailed	4	t: 5.880, P = 0.004
