# Supplementary material for: Aβ oligomer concentration in mouse and human brain and its drug-induced reduction ex vivo
Source: Cell Rep Med. 2022 May 17;3(5):100630. doi: 10.1016/j.xcrm.2022.100630 (PMC9133466; doi:10.1016/j.xcrm.2022.100630)
Supplement: Document S2. Article plus supplemental information [file mmc2.pdf]

Cell Reports
Medicine

Article

Aβ oligomer concentration in mouse and human brain and its drug-induced reduction *ex vivo*

Graphical abstract

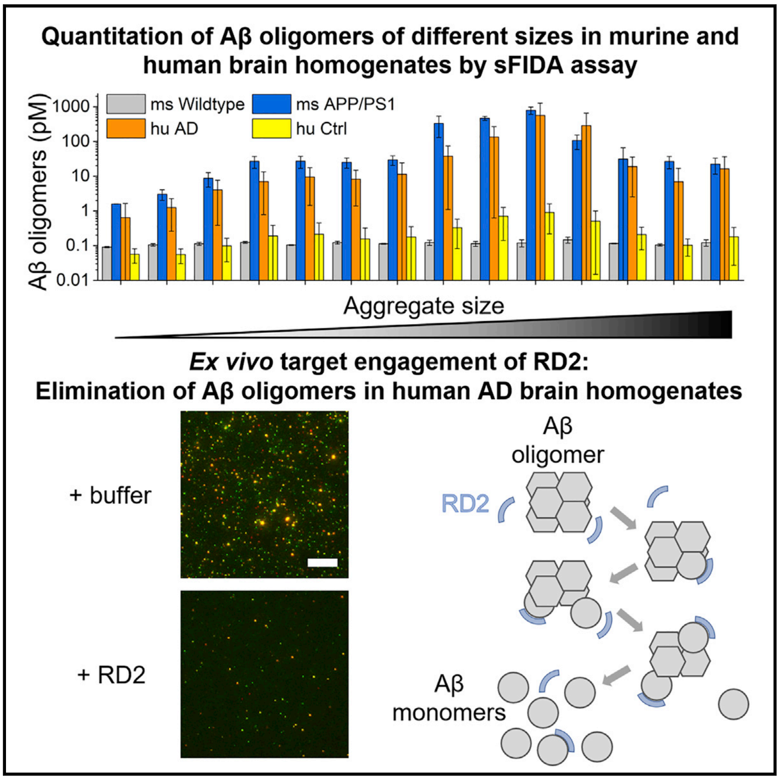

Highlights

- Quantitation of Aβ oligomers in murine and human brain homogenates by sFIDA assay
- Native Aβ oligomers from human AD brain homogenates are eliminated *ex vivo* by RD2
- Oligomer disassembly into monomers is incubation-time- and RD2-dose-dependent

Authors

Bettina Kass, Sarah Schemmert, Christian Zafiu, ..., Janine Kutzsche, Tuyen Bujnicki, Dieter Willbold

Correspondence

d.willbold@fz-juelich.de

In brief

Eliminating amyloid beta (Aβ) oligomers is a promising strategy for therapeutic drug development of Alzheimer's disease (AD). Here, Kass et al. quantitate Aβ oligomers in brain homogenates from various AD murine and human tissue samples and demonstrate the dose- and time-dependent disassembly of Aβ oligomers by the compound RD2.

Kass et al., 2022, Cell Reports Medicine *3*, 100630
May 17, 2022 © 2022 The Author(s).
https://doi.org/10.1016/j.xcrm.2022.100630

CellPress

Cell Reports Medicine

CellPress
OPEN ACCESS

Article

Aβ oligomer concentration in mouse and human brain and its drug-induced reduction *ex vivo*

Bettina Kass,[1] Sarah Schemmert,[1] Christian Zafiu,[1,3] Marlene Pils,[1,2,3] Oliver Bannach,[1,2,3] Janine Kutzsche,[1] Tuyen Bujnicki,[1] and Dieter Willbold[1,2,3,4,5,*]

[1]Institute of Biological Information Processing, Structural Biochemistry (IBI-7), Forschungszentrum Jülich, Jülich 52428, Germany
[2]Institut für Physikalische Biologie, Heinrich-Heine-Universität Düsseldorf, Düsseldorf 40225, Germany
[3]attyloid GmbH, Düsseldorf, 40225, Germany
[4]Priavoid GmbH, Düsseldorf, 40225, Germany
[5]Lead contact
*Correspondence: d.willbold@fz-juelich.de
https://doi.org/10.1016/j.xcrm.2022.100630

SUMMARY

The elimination of amyloid beta (Aβ) oligomers is a promising strategy for therapeutic drug development of Alzheimer's disease (AD). AD mouse models that develop Aβ pathology have been used to demonstrate *in vivo* efficacy of compounds that later failed in clinical development. Here, we analyze the concentration and size distribution of Aβ oligomers in different transgenic mouse models of AD and in human brain samples by surface-based fluorescence intensity distribution analysis (sFIDA), a highly sensitive method for detecting and quantitating protein aggregates. We demonstrate dose- and time-dependent oligomer elimination by the compound RD2 in mouse and human AD brain homogenates as sources of native Aβ oligomers. Such *ex vivo* target engagement analyses with mouse- and human-brain-derived oligomers have the potential to enhance the translational value from pre-clinical proof-of-concept studies to clinical trials.

INTRODUCTION

For 2020, the number of worldwide dementia cases was estimated to exceed 50 million,[1] with Alzheimer's disease (AD) being responsible for 60%–80% of all cases of dementia.[2] The disease's pathology is characterized by plaques consisting of amyloid beta (Aβ) fibrils in the extracellular space, neurofibrillary tangles composed of hyperphosphorylated tau protein fibrils inside neurons, and neurodegeneration. Still, no curative treatment of AD is available. There is agreement that by the time first cognitive symptoms become noticeable, the disease process has already been going on for decades.[3,4] Soluble oligomeric forms of Aβ are thought to be the most toxic species and have been described to be especially synapto- and neurotoxic.[5,6] Aβ oligomers are, therefore, a very attractive target for curative therapy approaches as well as for early diagnosis.

During the last years, we have developed compounds that are designed to stabilize Aβ monomers in their native, intrinsically disordered conformation. Thereby, the drug candidates destabilize Aβ oligomers and other Aβ assemblies and ultimately disassemble them directly into native Aβ monomers. In order to achieve this mode of action, we use all-D-enantiomeric peptides, which are known to be protease-resistant[7] and non-immunogenic.[8,9] The lead compound, D3, was selected by mirror-image phage display[10] and was shown to reduce Aβ aggregation and neuroinflammation and to improve cognition in a mouse model of AD even when applied orally.[11,12] Since then, numerous derivatives of D3 have been developed in order to optimize its binding properties and pharmacokinetic properties.[13–15] The most promising and clinically most advanced candidate is RD2. It is well characterized in terms of binding mode, target engagement, efficiency,[16–18] and pharmacokinetics.[19] Oral treatment with RD2 improved cognition in different mouse models of AD,[18,20] even in old-aged mice with full-blown pathology.[21] In the latter study[21], we demonstrated that RD2 treatment significantly reduced the concentration of Aβ oligomers, as measured by surface-based fluorescence intensity distribution analysis (sFIDA) in brain homogenates.

sFIDA realizes absolute specificity for Aβ aggregates over Aβ monomers. It achieves single aggregate particle sensitivity by combining the biochemical principle of a sandwich-ELISA with the readout of fluorescence intensity per pixel as obtained from fluorescence microscopy. Originally developed for the detection of prion protein aggregates,[22] sFIDA has been adapted for the quantitation of Aβ oligomers in cerebrospinal fluid (CSF)[23] and blood[24] and is in further development as a general tool for quantitating all possible protein aggregates.[25] sFIDA is specific for aggregates by using capture and detection antibodies that recognize overlapping or identical epitopes of the aggregated protein of interest. Mostly, two different fluorescence-labeled detection antibodies are used, and total internal reflection fluorescence (TIRF) microscopy images are recorded in both channels directly at the glass surface, providing superior single particle sensitivity compared with the ensemble signal used in ELISA-type assays. Only pixels above a certain intensity threshold that are co-localized in both channels are counted

© 2022 The Author(s).
This is an open access article under the CC BY license (http://creativecommons.org/licenses/by/4.0/).

(indicated as sFIDA readout), thereby ruling out possible unspecific signal of any of the used antibodies. Based on the sFIDA readout, concentrations can be calculated using a calibration standard, such as silica nanoparticles (SiNaPs) of a defined size, covalently coated with the capture and detection antibody-relevant epitopes.[26,27]

Here, we set out to characterize the amounts and the size distributions of Aβ oligomers in various amyloid-based animal models to compare them with each other and with human-brain-derived Aβ oligomers. Also, we demonstrate the usefulness of sFIDA to measure Aβ oligomer target engagement of the oligomer-eliminating compound RD2 in human brain homogenates. Such *ex vivo* target engagement based on patient-derived brain tissue (*ex vivo*) may well be suitable to enhance the translational value of pre-clinical *in vivo* experiments toward clinical trials.

RESULTS

Comparison of the concentrations of Aβ oligomers in density gradient centrifugation (DGC) fractions and in unfractionated brain homogenates from different mouse models of AD

Recently, sFIDA assay was adapted for quantitative detection of Aβ aggregates in complex matrices, such as brain homogenate, to demonstrate *in vivo* target engagement and validate the mechanism of action for RD2.[21] Brain homogenates were fractionated by density gradient centrifugation (DGC) prior to analysis by the sFIDA assay. In the current study, we analyzed specimens of three mouse models expressing different human APP variants based on human familial mutations in comparison with wild-type mice. Aβ oligomer concentrations were calculated based on SiNaP calibration standards and are displayed in Figure 1A as concentrations in undiluted brain homogenate or DGC-obtained fractions, resulting in an apparent concentration of 1 pM in unfractionated wild-type brain homogenate. The average sFIDA readout observed in wild-type DGC fractions barely exceeded that of the buffer control. The antibodies IC16 and Nab228 used in this assay are specific for human Aβ, which is absent in wild-type mice. This outcome matched the expectations for wild-type mice as a negative control. Wild types were, therefore, not included in the calculation of relative oligomer concentrations, shown in Figure 1B. Western blot analysis performed with antibody 6E10, which is also specific for human Aβ, also did not yield specific bands in wild-type DGC fractions, as displayed in Figure 1C.

In transgenic mouse samples, the highest Aβ oligomer titers were found in fractions 9 (APP_{SwDI}) or 10 (APP/PS1 and APP_{Lon}), with mean concentrations of up to 16.0 ± 9.7 pM in APP_{SwDI}, 790 ± 190 pM in APP/PS1, and 300 ± 150 pM in APP_{Lon} samples. Samples showed high inter-individual heterogeneity, and the respective peak fractions made up 26.6% ± 16.2% (APP_{SwDI}), 41.8% ± 9.9% (APP/PS1), or 30.3% ± 14.5% (APP_{Lon}) of all oligomers measured in DGC fractions of the respective mouse models Figure 1B. A local maximum was identified in all transgenic mouse models, covering fractions 4 and 5. The amounts of oligomers found in these two fractions together made up 13% (APP_{SwDI}), 2.8% (APP/PS1), and 6.9% (APP_{Lon}) of the total oligomer concentration. In this type of density gradient, calibrated with globular proteins, fractions 4 and 5 would correspond to a size of 66–150 kDa, and fractions 9 and 10 to a size of at least 400 kDa, respectively.[28] The overall distribution of total Aβ detected via western blot with antibody 6E10, shown in Figure 1C, was similar to the sFIDA results of the different mouse models. However, APP_{SwDI} Aβ bands were of equal or higher intensity than those of the other two mouse models, although the Aβ oligomer concentration measured by sFIDA in this mouse model was substantially lower. All three antibodies worked equally well in western blot (Figure S1), so major differences in the general detectability of denatured DI-Aβ by IC16 and Nab228 in comparison with 6E10 were ruled out as a possible reason for this observed difference. Recovery was calculated as the ratio of the total amount of oligomers measured by sFIDA assay in all fractions to the total amount of oligomers in the corresponding unfractionated homogenates. The respective recovery rates were 0.986 for APP_{SwDI}, 1.634 for APP/PS1, and 0.975 for APP_{Lon} samples. Overall, this shows that the chosen dilutions of 1:10 for DGC fractions and 1:100 for 10% brain homogenates were well suited for quantitation of oligomers in APP_{SwDI}, APP/PS1, and APP_{Lon} mice, while applying less diluted wild-type samples did not cause any artifacts. Small amounts of sample would therefore be sufficient to investigate *in vivo* target engagement of oligomer-eliminating compounds in several different mouse models, in a similar fashion to Schemmert et al.[21]

Notable differences of the concentration of Aβ oligomers between human AD samples and non-demented controls

The previously analyzed transgenic mouse models of AD play an important role in the development of therapeutic compounds that would ultimately be used in human patients. In order to mimic the clinical situation more accurately, we investigated the concentrations and size distributions of Aβ oligomers in *post mortem* brain homogenates of human AD patients as well as in age-matched, non-demented control subjects (NCs). Details of human brain donors can be found in Table 1. While the four NCs had similar oligomer concentrations, the AD samples showed large differences between each other, as indicated in Figures 2A and 2B. In general, the Aβ oligomer concentration in the NC group was more than 10-fold lower than the lowest oligomer concentration in the AD group but clearly exceeding the limit of detection (LOD) of 7.6 fM and lower limit of quantitation (LLOQ) of 9.7 fM With the exception of sample AD3, DGC fraction 10 contained the highest Aβ oligomer concentrations, ranging from 19.9 ± 3.8 pM (AD1) to 1820 ± 191 pM (AD6) and 0.9 ± 0.7 pM in NC samples. These oligomer particles, corresponding to a calibrated size of more than 450 kDa,[28] made up 33.9% ± 6.5% to 54.5% ± 5.7% in all AD samples, except for AD3, and 23.4% ± 1.8% in NC samples, as depicted in Figure 2C. In sample AD3, the highest oligomer concentration (50.3 ± 9.1 pM) was found in fraction 11 and a concentration of 42.7 ± 1.1 pM in fraction 10, which corresponded to 33.8% ± 6.1% and 28.6% ± 0.3% of all oligomers, respectively. Similar to the observations in transgenic mouse samples, local maxima were identified. A local maximum was found in fraction 5 in almost all

CellPress
OPEN ACCESS

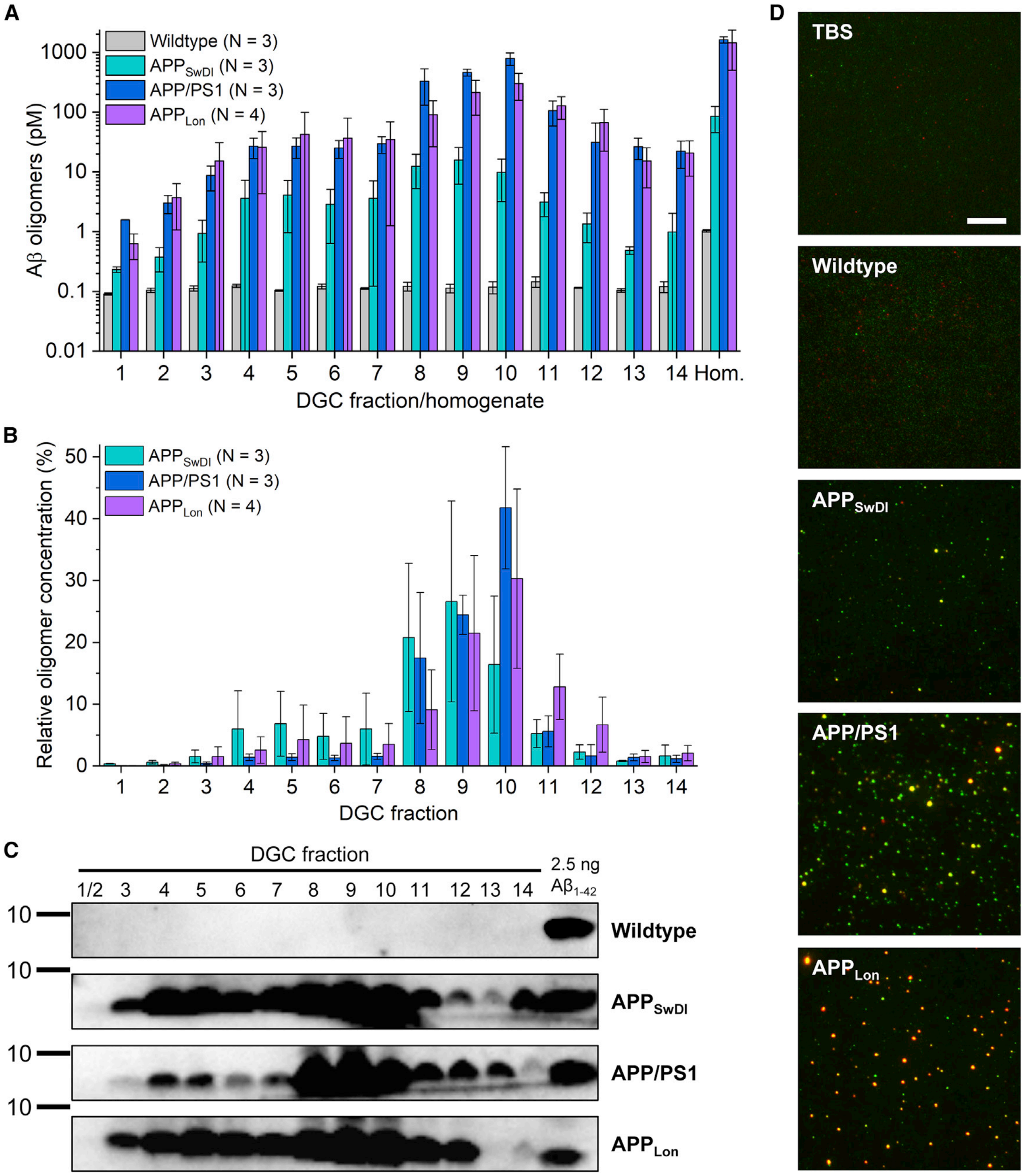

Figure 1. Comparison of the concentrations of Aβ oligomers in DGC-obtained fractions and in unfractionated brain homogenates of different mouse models of AD

(A) Overall distribution of different Aβ oligomers separated by DGC in different mouse models measured by sFIDA. Concentrations are displayed as they were present in DGC fractions or 10% brain homogenate (Hom.), respectively, before any dilution steps during the sFIDA experiments. Please note the logarithmic scale of the y axis.

(B) Relative concentration in percent of the total concentration of all fractions of oligomers in transgenic mouse samples. See Figure S1F for individual data points, referring to Figure 1B. Data in (A) and (B) are presented as mean ± SD of the indicated number (N) of animals.

(C) Western blot analysis of undiluted DGC fractions (12 μL per lane) of one representative animal per group in comparison with a synthetic $A\beta_{1-42}$ standard showing Aβ bands detected by monoclonal antibody 6E10. See also Figure S1 for full blots.

(D) Representative sections of TIRF images of brain homogenate samples from transgenic mice and from wild-type mice as well as from a buffer control. Final dilution factors of APP/PS1, APP_{Lon}, and APP_{SwDI} mouse brain homogenates were 1:200; wild-type mouse brain homogenates are shown at a final dilution of 1:20. Red and green fluorescence channels were merged, depicting co-localized (yellow) pixels used for calculating the oligomer concentration. The scale bar represents 10 μm.

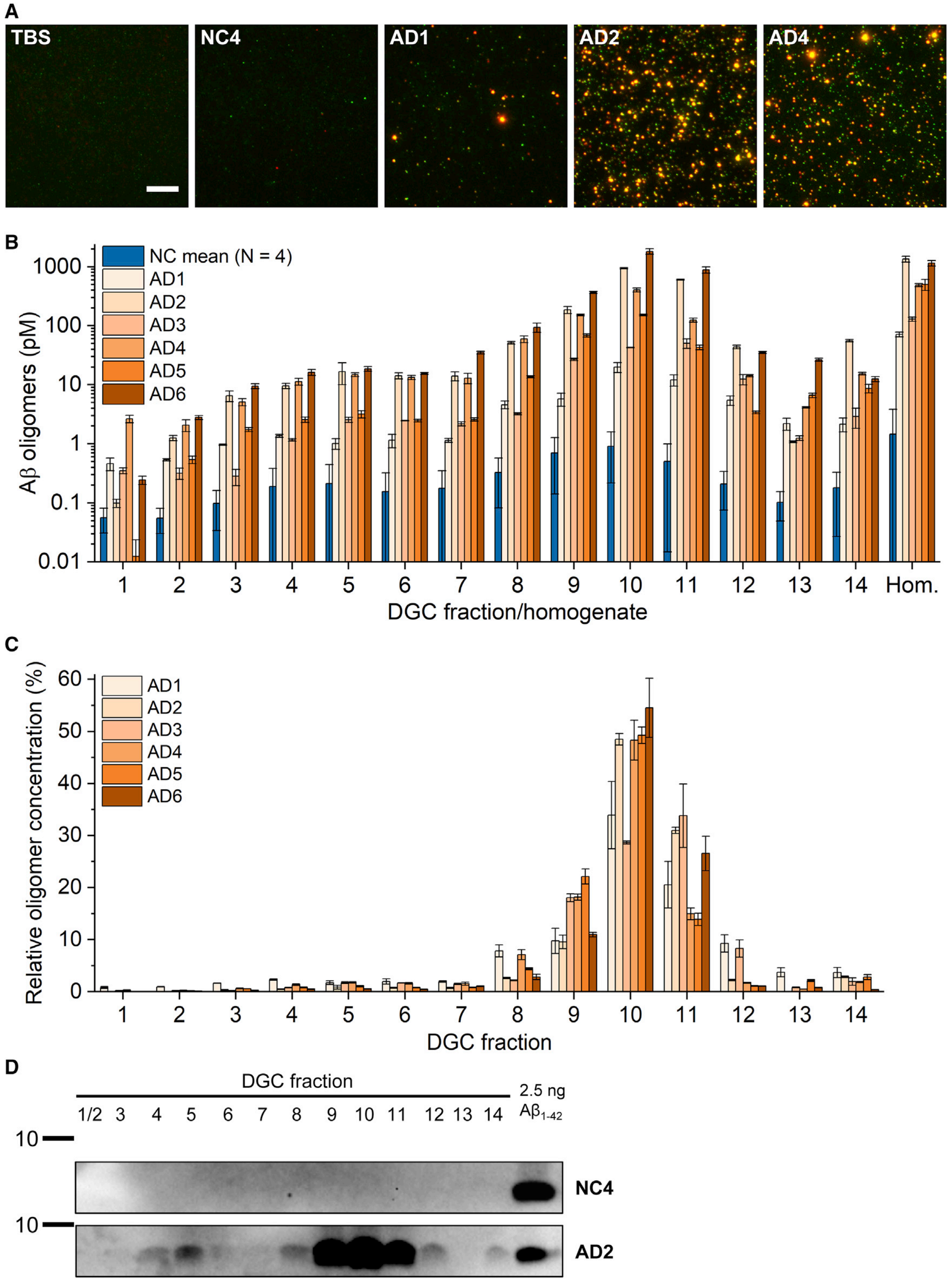

Figure 2. Notable differences of the concentration of Aβ oligomers between human AD samples and non-demented controls

(A) Representative sections of TIRF images of unfractionated human brain homogenates at a final dilution of 1:10. A merged image of red and green fluorescence channels is shown, depicting co-localized (yellow) pixels used for calculating the oligomer concentration. The scale bar represents 10 μm.

(B) Overall distribution of different Aβ oligomers separated by DGC measured by sFIDA. Concentrations are displayed as they were present in undiluted DGC fractions or 10% brain homogenate, respectively, before any dilution steps during the sFIDA experiments. Please note the logarithmic scale of the y axis.

(legend continued on next page)

samples, AD1 being the only exception, with a local maximum in fraction 4. The size of these oligomers agrees well with the size of artificially prepared Aβ oligomers that have not yet elongated and do not contain other components besides Aβ.[28] The percentage of total oligomers found in the respective local maximum fraction was in the range of 0.6% ± 0.1% to 2.3% ± 0.1% for AD samples and 5.5% ± 0.6% for NC samples. In a corresponding western blot of total Aβ, no bands were detectable in the NC2 sample, with the distribution of total Aβ across the density gradient matching the sFIDA results for the AD4 sample (Figure 2D; uncropped western blot images can be found in Figure S2). Recovery rates were 1.15, 2.02, 1.61, 2.40, 0.86, and 4.06 for AD1 to AD6, respectively. Here, the sFIDA assay has demonstrated its usefulness to determine concentrations of Aβ oligomers, even in complex samples like brain homogenates with a wide range of oligomer particle sizes. While the diversity of oligomer concentrations found in human samples was much larger compared with transgenic mice, the overall size distribution was very similar to that of aged APP/PS1 mice. Recovery rates exceeding 1 by a larger margin could be due to underestimation of the oligomer concentration in unfractionated brain homogenates. This effect occurred mostly in the three AD samples with the highest oligomer concentration. As a consequence, greater dilution factors of unfractionated homogenate have been used in the following experiments to reduce the possible influence of other proteins and to avoid saturation effects, while still staying in the quantifiable, linear range of the assay.

Ex vivo treatment with RD2 results in a dose- and incubation-time-dependent reduction of Aβ oligomers derived from APP/PS1 mouse or human brain homogenate with RD2

The all-D-enantiomeric compound RD2 has been developed to stabilize Aβ monomers in their native conformation, thus destabilizing Aβ assemblies and ultimately disassembling them into Aβ monomers. RD2 has been shown to improve cognition in several AD mouse models,[18,20,21] and its oligomer-eliminating effect has been well characterized using synthetic Aβ.[17,18] RD2 did not display any toxic effects in mice or in cell cultures, indicating that the possible disruption of HMW oligomer species by RD2 did not produce smaller, toxic oligomer species but non-toxic monomers.[18,21] In a study conducted in APP/PS1 mice, a reduction of Aβ oligomer concentration was measured by the sFIDA assay in brain homogenates of RD2-treated mice, demonstrating *in vivo* target engagement and suggesting elimination of these oligomers by RD2.[21] That study showed that oligomers were significantly reduced in brain homogenates of APP/PS1 mice after oral treatment with RD2 in comparison with placebo. DGC fraction 10 contained the highest concentration of Aβ oligomers. We therefore first investigated the effect of RD2 on this isolated fraction 10 in an *ex vivo* approach. RD2 or buffer was added to 1:2 diluted fraction 10 of APP/PS1 mouse brain homogenate, and samples were drawn after the indicated incubation times. In cases of "0 min" incubation time, samples were mixed and immediately flash-frozen. As shown in Figure 3, RD2 was able to reduce the concentration of oligomers in a dose- and time-dependent manner. After the maximum incubation time of 20 h, an 81% reduction of oligomers was achieved with 50 μM, and 67% reduction was observed with 20 μM. The incubation time dependence clearly indicates that the RD2 dose dependence of oligomer reduction is not due to competition with the detection antibodies. A slight effect could also be seen with 10 μM RD2, yielding a reduction of about 8% after 20 h, but this change was not statistically significant. It must be noted that samples incubated with 20 and 50 μM RD2 showed a remarkably reduced oligomer concentration even without additional incubation time (Figure 3B, 0 min): the baseline oligomer concentration of the sample incubated with buffer was 88 ± 6 pM, whereas samples with the addition of 20 or 50 μM RD2 had Aβ oligomer concentrations of 48 ± 3 pM and 36 ± 2 pM, respectively. A possible explanation could be an ongoing reaction of RD2 with oligomers during sample incubation on the capture surface, thereby prolonging the effective reaction time by about 2 h. Another possible explanation would be that the initial elimination of oligomers is a fast process and that the delay of several minutes during the sample preparation steps due to freezing and thawing the samples before analysis would make observation of a true baseline value difficult. To address these questions and in order to rule out any delays during sample preparation, freeze-thaw cycles were omitted in further experiments involving short incubation times and 0 min marks. The dose-dependent reduction of Aβ oligomers from APP/PS1 mouse brain strongly suggests successful *ex vivo* target engagement of RD2.

Next, we wanted to investigate whether RD2 shows similar *ex vivo* target engagement on Aβ oligomers in brain homogenates derived from AD patients. Based on the aforementioned sFIDA analysis of several human brain homogenates (Figure 2), homogenate sample AD2 was chosen for further analysis, because this sample had one of the largest quantities of Aβ aggregates, allowing robust detection of signal across a range of dilutions, even considering the possible drastic reduction of sFIDA signal by the addition of RD2. The 10% homogenate sample was serially diluted before being incubated with different concentrations of RD2, ranging from 0.31 to 20 μM. In addition to buffer as a negative control, we chose another all-D-enantiomeric compound of similar size, D1, as a negative control peptide. D1 was originally selected for binding Aβ fibrils, but not for specific elimination of oligomers,[29] and was further developed as a positron emission tomography (PET) tracer rather than a therapeutic compound.[30–32] After an incubation time of 24 h, samples were subjected to sFIDA assay, and concentrations were calculated with SiNaP standards. Refer to Figure S3A for representative TIRF images. The 1:10 diluted brain homogenate that was incubated only with buffer, shown in Figure 4A, had a concentration of 47.1 ± 20.0 pM Aβ oligomers. Samples incubated with D1 showed an

(C) Relative concentration in percent of the total concentration of all fractions of oligomers in human brain homogenates fractionated by DGC. Data in (B) and (C) are presented as mean ± SD AD samples (N = 6, separately shown for each AD sample): N = 3 (technical replicates); NC samples: N = 4 (biological replicates).
(D) Exemplary western blot analysis of undiluted DGC fractions (12 μL per lane) in comparison with a synthetic $A\beta_{1\text{-}42}$ standard showing Aβ bands detected by monoclonal antibody 6E10. Marker bands indicate 10 kDa. See also Figure S2 for full blots.

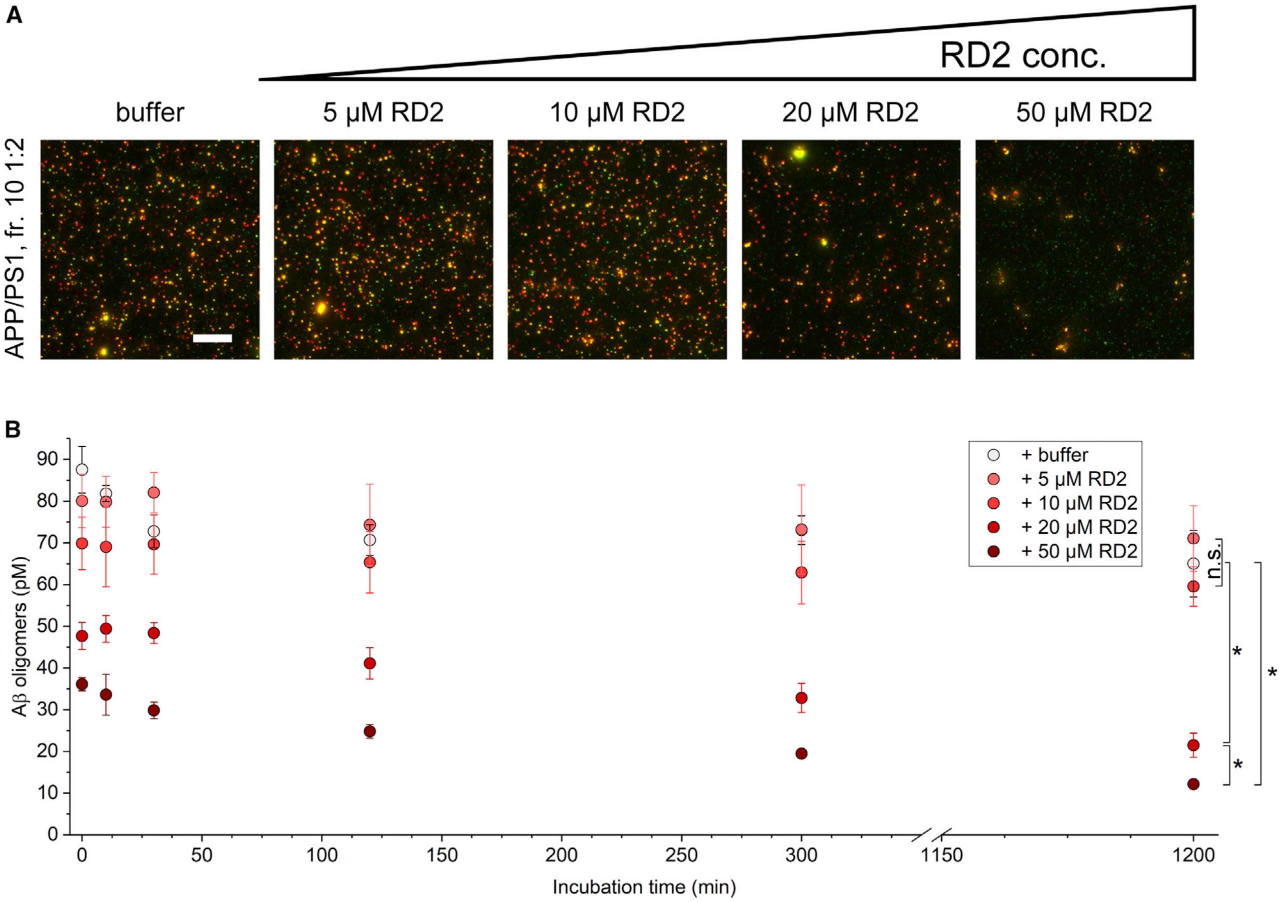

Figure 3. Dose- and incubation-time-dependent effect of RD2 on fractionated APP/PS1 mouse brain homogenate

DGC fraction 10 of APP/PS1 mouse brain homogenate, representing the peak of sFIDA signal, was diluted 1:2 and was incubated with 0, 5, 10, 20, or 50 μM RD2. Before analysis, samples were further diluted 1:5, resulting in a total dilution of 1:10, and the Aβ oligomer concentration was immediately analyzed by sFIDA assay. The final dilution factor during image acquisition was 1:20.

(A) Sections of representative TIRF images after 20 h of incubation. Red and green fluorescence channels were merged. The scale bar represents 10 μm.

(B) Aβ oligomer concentrations present in the original 1:2 diluted sample. Concentrations given here reflect the actual concentrations in the prepared sample after dilution and are not directly related to the concentrations in undiluted samples, shown in Figure 1.

Data are presented as mean ± SD *between groups, $p < 0.05$ (Kruskal-Wallis one-way ANOVA on ranks with Student-Newman-Keuls *post hoc* analysis). N = 3 (technical replicates).

overall reduced concentration of 30.2 ± 4.1 pM to 37.1 ± 3.7 pM, but no dose-dependent effect was observed. The same could be observed for the three lowest concentrations of RD2. The addition of 20 μM RD2 resulted in a drastic, significant reduction of the Aβ oligomer concentration down to 3.3 ± 0.4 pM, corresponding to a reduction by 93% compared with the buffer control. The Aβ oligomer concentration in 1:20 diluted homogenate was 12.8 ± 1.8 pM in the sample incubated with buffer (Figure 4B). Addition of D1 of any concentration or of 0.31 and 1.25 μM RD2 resulted in a similar reduction as observed in the 1:10 diluted homogenate sample, with no significant dose-dependent effect. Effects of 5 and 20 μM RD2 were distinct, with a reduction of oligomers to 4.9 ± 1.0 pM (61%) and 0.6 ± 0.1 pM (95%), respectively. The results for 1:40 diluted brain homogenate are shown in Figure 4C: after 24 h incubation with 5 and 20 μM RD2, the concentration of Aβ oligomers was reduced to 0.7 ± 0.1 pM (84%) and 0.2 ± 0.04 pM (96%) from 4.1 ± 1.1 pM. A tendency of oligomer reduction was also observed with 0.31 and 1.25 μM RD2, with a reduction to 2.7 ± 0.1 pM (36%) and 2.1 ± 0.1 pM (50%), respectively, of which only 1.25 μM RD2 caused an oligomer reduction that was significantly different from that observed with all concentrations of the control peptide D1. Different degrees of signal reduction were observed with the control peptide D1, ranging from 4% to 27% with no evidence of a dose-effect. Despite a certain reduction of Aβ oligomers in D1-treated samples, this effect was not dose-dependent, leading to the conclusion that only RD2 showed a specific reduction effect on Aβ oligomers in human AD brain homogenate.

Time- and dose-dependent elimination of oligomers in human brain homogenate by RD2 but not by control D-peptides D1 and QB37

To gain more insight into the dynamics of the *ex vivo* target engagement of RD2 in human AD brain homogenate, we

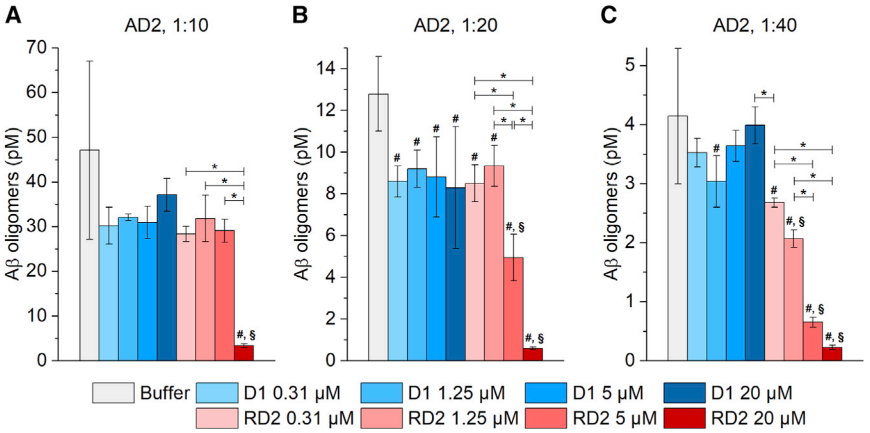

Figure 4. Dose-dependent reduction of Aβ oligomer concentration in human brain homogenate incubated with RD2

Human AD brain homogenate (sample AD2) was diluted 1:10, 1:20, or 1:40 and was incubated with 0.31, 1.25, 5, or 20 μM (factor of four dilution scheme) of D-peptides RD2 or D1 for one day (24 h).

(A–C) D1 was used as a control peptide. Samples were measured by (aggregate-specific) sFIDA assay, and the concentration of Aβ oligomers was calculated based on SiNaP standards. Results for dilutions of 1:10, 1:20, and 1:40 are displayed in (A), (B), and (C), respectively. Concentrations given here reflect the actual concentrations in the prepared sample after dilution and are not directly related to the concentrations in undiluted samples, shown in Figure 2. Data are presented as mean ± SD N = 3 (technical replicates) *between groups, $p < 0.05$; #versus buffer, $p < 0.05$; §versus all concentrations of D1, $p < 0.05$ (one-way ANOVA with Student-Newman-Keuls *post hoc* analysis). The corresponding representative TIRF images are found in Figure S3.

monitored the Aβ oligomer concentration in diluted homogenates from four different AD patients with incubation times ranging from 0 to 23 h. These samples were chosen because of their high concentration of Aβ oligomers, as determined previously, to allow monitoring of changes over a wide signal range. In addition to the previously tested peptide D1, QB37 was added as an additional control D-peptide of similar size to RD2 and D1. The homogenate was used at a dilution of 1:20; both control peptides were used at a concentration of 20 μM; and RD2 concentrations were 1.25, 5, and 20 μM. Figures 5A, 5C, 5E, and 5G show baseline concentrations measured by sFIDA assay after 0 min incubation time and endpoint concentrations after 1,365 min (AD2); 1,3,57 min (AD4 and AD5); or 1,425 min (AD6) of incubation; for representative TIRF images of samples AD2 and AD6 at their endpoints, see Figure S3B. At baseline, none of the peptides, including RD2, caused a reduction of Aβ oligomers in comparison with the buffer controls. The design of sFIDA assay includes an incubation time of at least 1.5 h after the indicated pre-incubation times. Without any pre-incubation time (0 min), no sFIDA signal reduction was observed. This indicates that the signal reduction observed after longer incubation times was attributed to the reaction of the Aβ oligomers with RD2 but that no additional reaction took place once the oligomers were captured on the sFIDA plate. After approximately 23 h of incubation, samples incubated with 5 and 20 μM RD2 showed a significant decrease in oligomer concentration to 1.0 ± 0.3 pM (57% decrease) and 0.1 ± 0.1 pM (93%) for AD2, 0.41 ± 0.03 pM (81%) and 0.19 ± 0.03 pM (91%) for AD4, 0.44 ± 0.05 pM (73%) and 0.18 ± 0.02 pM (89%) for AD5, or 3.6 ± 0.4 pM (64%) and 1.0 ± 0.2 pM (90%) for AD6 compared with the buffer control. Results for additional incubation times of these two RD2 concentrations are shown in Figures 5B, 5D, 5E, and 5H, demonstrating a rapid decrease of oligomer concentration within the first 40 to 50 min of the reaction with 20 μM RD2, followed by a considerably slower further reduction of the oligomer concentration in the following hours. In order to describe our observations using a global kinetic fit based on a pseudo-first-order reaction, a double exponential decay function was used, essentially reflecting a combination of two reactions taking place at different rates. Importantly, a threshold concentration of RD2 was assumed for these calculations, because an effect was observed only with a dose of 5 μM but not with 1.25 μM RD2 or lower. Due to possible binding of RD2 to various other components of brain homogenate, the effective RD2 concentrations were expected to be considerably smaller than the total concentration. The global fits shown in Figures 5B, 5D, 5F, and 5H are therefore based on a threshold concentration of 4 μM, meaning that the remaining effective RD2 concentrations were 1 and 16 μM instead of 5 and 20 μM, respectively. Reaction rate constants $k_{1,fast}$ of 2,329; 2,928; 2,374; and 1,275 $L*mol^{-1}*min^{-1}$ were calculated for AD2, AD4, AD5, and AD6, respectively. The reaction rate constants $k_{1,slow}$ were 76, 37, 50, and 48 $L*mol^{-1}*min^{-1}$ for AD2, AD4, AD5, and AD6. Similar to our previous observation, shown in Figure 4, with samples from AD2, three of the samples showed a reduction of Aβ oligomers with 20 μM D1 as well. The effect was much weaker than with 5 μM RD2 and is most likely not specific, due to the absence of a dose-dependent effect, as stated before (Figure 4).

Effect of RD2 on different DGC fractions of human brain homogenate

Fractions 5 and 10 of AD brain homogenate were identified as local and total Aβ oligomer concentration peak fractions, indicating distinct sizes and possibly different types of aggregates. To investigate the effect of RD2 on these fractions while staying in the quantifiable concentration range of the assay, fraction 10 was diluted 1:10, and fractions 4 to 6 were pooled with no further dilution. While fractions 4 to 6 would possibly represent oligomers with sizes similar to that of synthetic Aβ oligomers in absence of other proteins, in fraction 10, larger Aβ assembly species are present that possibly also consist of additional proteins and form co-aggregates. Fraction 10 was of particular interest because the reduction of oligomers found in this fraction correlated with improved cognition in transgenic mice treated with RD2.[21] For incubation with fraction 10, RD2 was used at 5 and 20 μM, and pooled fractions 4 to 6 were incubated with 20 μM

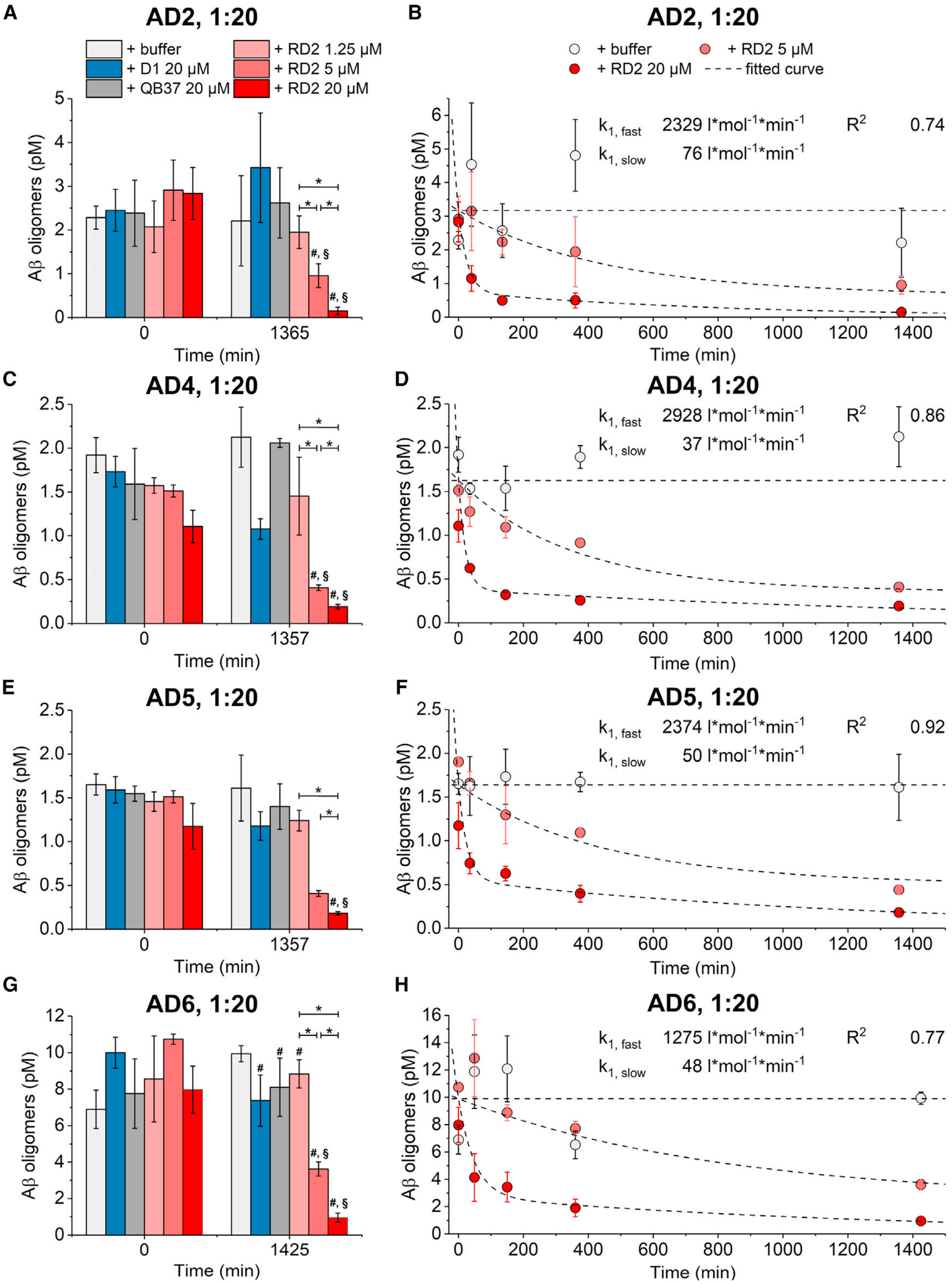

Figure 5. Time- and dose-dependent elimination of oligomers in human brain homogenate by RD2 but not by D-peptides D1 and QB37

Human AD brain homogenate (AD cases 2, 4, 5, and 6) was diluted 1:20 and was incubated with different concentrations of RD2 or 20 μM D1 or QB37. After incubating for the indicated amounts of time, samples were analyzed by sFIDA assay.

(A–H) Aβ oligomer concentrations at baseline and after overnight incubation are displayed for AD2 (A), AD4 (C), AD5 (E), and AD6 (G). Concentrations given here reflect the actual concentrations in the prepared sample after dilution and are not directly related to the concentrations in undiluted samples, shown in Figure 2.

(legend continued on next page)

RD2. sFIDA results for fraction 10 are shown in Figures 6A and 6B for samples AD2 and AD6; representative TIRF images are shown in Figure S4A. After 0 min of pre-incubation, baseline concentration of fractions without peptides was around 11 pM for AD2 and 8 pM for AD6. A slight decrease in concentration of these samples was observed after 327 and 1,380 min to around 9 pM (AD2) and 6 pM (AD6). In contrast to the previous findings on unfractionated homogenates, fraction 10 of both samples showed a reduction of oligomer concentration by approximately 50% already at 0 min pre-incubation time with 5 μM RD2 and over 60% with 20 μM RD2. The concentration of Aβ oligomers in RD2-treated samples decreased further over the course of 1,380 min, down to 1.7 ± 0.1 pM (5 μM RD2, 81%) and 0.7 ± 0.1 pM (20 μM RD2, 92%) in AD2 and 1.4 ± 0.1 pM (5 μM, 77%) and 0.7 ± 0.1 pM (20 μM, 88%) in AD6. With 20 μM RD2, the endpoint values were comparable with those found in unfractionated homogenate, whereas 5 μM RD2 had an overall slightly larger effect on fraction 10 than on homogenate. The time- and dose-dependent response observed here with DGC-derived fraction 10 of human AD brain homogenate as well as the seemingly instant reduction of oligomers without additional pre-incubation time is very similar to the one observed in fraction 10 of APP/PS1 mouse brain homogenate before (Figure 3). The pooled fractions 4 to 6 showed low baseline values of 0.35 ± 0.03 pM (AD2) and 0.51 ± 0.04 pM (AD6), as presented in Figures 6C and 6D (Figure S4B: representative TIRF images). Baseline concentrations were identical for the samples incubated with buffer and samples with 20 μM RD2. After 1,380 min, the concentrations of the samples incubated with 20 μM RD2 were notably reduced to 0.12 ± 0.01 pM (AD2, 64%) and 0.14 ± 0.01 pM (AD6, 62%).

DISCUSSION

Size distribution of Aβ aggregates in relevant AD mouse models and patient-derived samples

Transgenic mouse models of AD are commonly used for understanding disease development and testing potential disease-modifying drugs. However, there is no single mouse model of choice that displays the complete and holistic pathology of AD, including Aβ plaques, tangles, neurodegeneration, and cognitive decline. Therefore, several different mouse models are used to investigate the efficacy of potential drug candidates. We analyzed the Aβ oligomer size distributions in brain homogenates of aged specimens of three different mouse models of AD and human AD cases, using the aggregate-specific sFIDA assay in combination with DGC. Our choice of extraction and analysis methods was intended to keep all aggregates as native as possible. In general, a majority of Aβ aggregates were found in fractions 9 or 10, corresponding to a calibrated size of more than 400 kDa in all transgenic mice and in human AD as well as NC samples. Aβ aggregates of similar size have been reported in transgenic mice and human AD cases before, using methods, such as size-exclusion chromatography (SEC), blue native blots, or density gradients. They have been found to have oligomeric or protofibrillar conformation[33–35] and have been postulated to serve as a reservoir for smaller, more diffusible toxic oligomers rather than being toxic themselves.[36,37] Fractions 4 to 6 were of special interest because they contained especially neurotoxic oligomers between 66 and 150 kDa in experiments with synthetic Aβ[28] and presented a local maximum in all transgenic mice and human samples. Oligomers in this size range might therefore have particular biological significance despite their lower relative concentration in the samples.

It is also likely that different co-aggregates of Aβ are found in different fractions. Oligomer binding proteins, such as apolipoprotein E (ApoE), influence the apparent aggregate size of Aβ assemblies consisting of otherwise similar numbers of Aβ units.[38] The investigation of the composition and function of potential co-aggregates in these fractions is a subject of further research.

In comparison with the strong bands observed in the western blot using the antibody 6E10, the concentrations of Aβ oligomers from APP_{SwDI} mouse brains measured by sFIDA assay using the antibodies Nab228 and IC16 were lower than expected, albeit still within the dynamic range of the assay. A control of the western blot using antibodies Nab228 and IC16 also yielded strong bands (Figure S1). These findings suggest that Aβ oligomers from APP_{SwDI} brain homogenate samples have less accessible epitopes for the antibodies used in the sFIDA but that these epitopes were released by the denaturing conditions of the SDS-PAGE. In contrast to APP_{Lon} and APP/PS1 mice, the Aβ variant expressed in APP_{SwDI} mice contains two amino acid residue replacements (E22Q/D23N). This highly artificial APP variant, which does not exist in humans, possibly results in Aβ monomers that form oligomers with conformations that are different from those obtained from other transgenic models and humans. Such different conformations possibly result in reduced accessibility of the epitopes for the respective antibodies in non-denaturing conditions, even though the epitopes as such are not affected by the APP mutations. Still, we were able to clearly discriminate APP_{SwDI} samples from wild-type samples in all fractions at a 1:10 dilution. All transgenic mice tested here showed full-blown pathology, which would be relevant for curative studies like the one conducted by Schemmert et al.,[21] in which sFIDA assay was used for the first time to monitor *in vivo* target engagement. The development and possible changes in aggregate size can be monitored in mice of different ages to further characterize existing or novel mouse models of AD.

Data are presented as mean ± SD N = 3 (technical replicates). *between groups, $p < 0.05$; #versus buffer, $p < 0.05$; §versus both control peptides D1 and QB37, $p < 0.05$ (Kruskal-Wallis one-way ANOVA on ranks with Student-Newman-Keuls *post hoc* analysis). (B), (D), (F), and (H) Determination of the reaction rate constants $k_{1,fast}$ and $k_{1,slow}$ by a global double exponential fit of AD2 (B), AD4 (D), AD5 (F), or AD6 (H). Only the data corresponding to the three RD2 concentrations that were significantly different from each other (0 [buffer], 5, and 20 μM) were used. Effective RD2 concentrations were assumed to be 1 μM for the 5 μM data and 16 μM for the 20 μM data. Concentrations given here reflect the actual concentrations in the prepared sample after dilution and are not directly related to the concentrations in undiluted samples, shown in Figure 2. Data are presented as mean ± SD N = 3 (technical replicates). Refer to Figure S3 for representative TIRF images and to Figure S6 for the proposed mechanism of oligomer elimination by RD2.

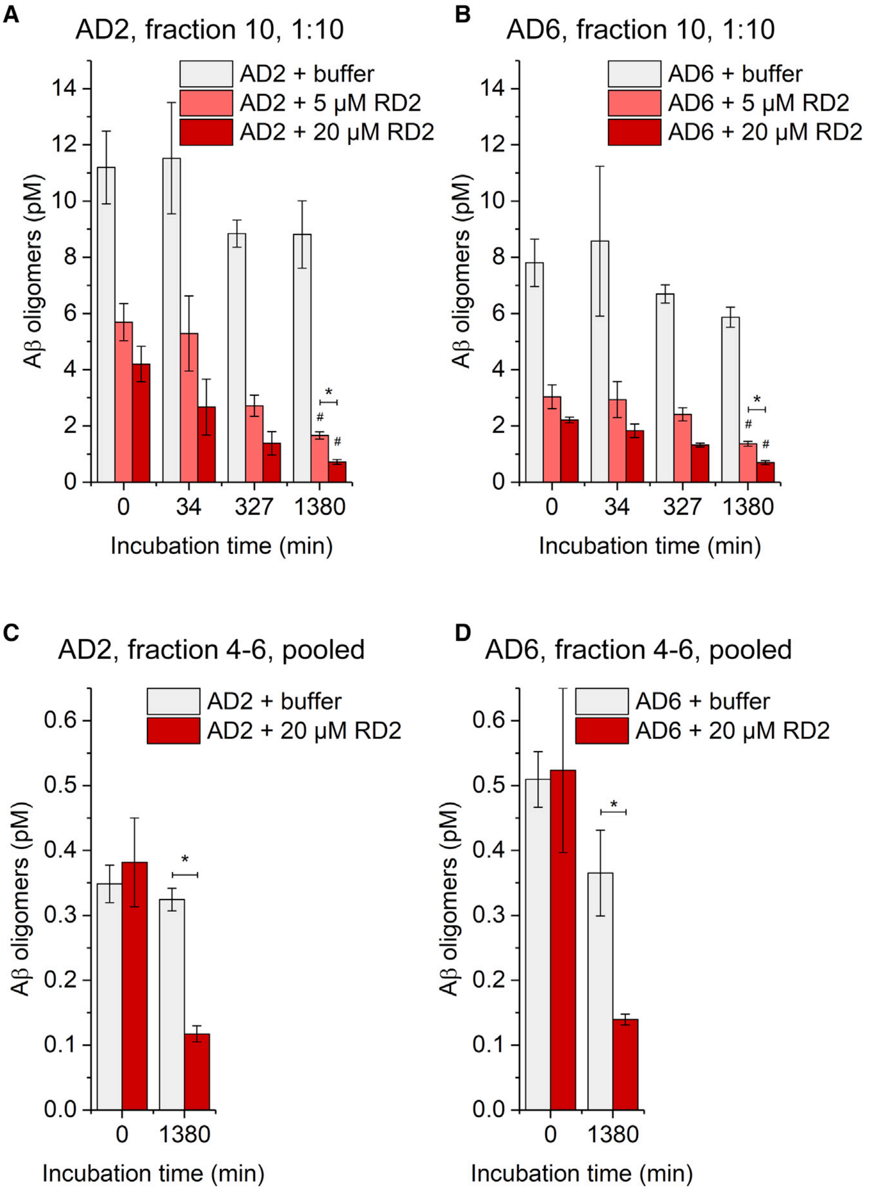

Figure 6. Effect of RD2 on different DGC fractions of human brain homogenate

(A and B) DGC fraction 10 of human AD brain homogenate (AD cases 2 and 6), representing the peak of sFIDA signal, was diluted 1:10 and was incubated with 0, 5, or 20 μM RD2. After incubating for the indicated duration, the Aβ oligomer concentration of the samples was analyzed by sFIDA assay. Concentrations given here reflect the actual concentrations in the prepared sample after dilution and are not directly related to the concentrations in undiluted samples, shown in Figure 2. Data are presented as mean concentration ±SD N = 3 (technical replicates). *between groups, $p < 0.05$; #versus buffer, $p < 0.05$ (Kruskal-Wallis one-way ANOVA on ranks with Student-Newman-Keuls *post hoc* analysis).

(C and D) DGC fractions 4 to 6 of human AD brain homogenate (AD cases 2 and 6), representing a local maximum in the size distribution of Aβ oligomers, were pooled and were incubated with 0 or 20 μM RD2 overnight. Samples were analyzed by sFIDA assay. Concentrations given here reflect the actual concentrations in the prepared sample after dilution and are not directly related to the concentrations in undiluted samples, shown in Figure 2. Data are presented as mean ± SD N = 3 (technical replicates). *between groups, $p < 0.05$ (two-tailed t test); TIRF images are displayed in Figure S4.

Human AD brain homogenates showed much larger inter-individual variability of their total Aβ oligomer concentrations compared with those of transgenic mice, which was expectable due to the generally larger variation among human patients compared with inbred mouse strains. It is also possible that the location from which the samples were taken for homogenization affected the total amount of aggregates later found in the sample: All human samples used in the present study were from the superior parietal lobule, but inhomogeneity of the local distribution of Aβ might still occur, which would not be noticeable in mice of which the whole brains were used. Although mostly lacking Aβ pathology, NC samples also showed low amounts of oligomers with a maximum in fraction 10 as well. The occurrence of these miniscule amounts of oligomers is in line with the fact that sporadic accumulation of Aβ can generally be found long before cognitive symptoms would occur, if the afflicted person were to develop AD at all.[39] Regardless of the differences in absolute concentrations and in amyloid plaque pathology, the relative distribution of Aβ oligomer in AD samples was mostly comparable with that of APP/PS1 mice, possibly indicating similar types of aggregates.

Ex vivo target engagement of the oligomer-eliminating compound RD2

There is an urgent need to assess the value of pre-clinical animal experiments for their predictability in clinical studies, especially in the field of AD. Previously, we described that oral treatment with RD2 in aged APP/PS1 mice with full-blown pathology yielded improvement of cognition, memory, and behavior. We rationalized that this outcome was based on the efficacy of the compound to directly eliminate toxic Aβ oligomers, a mode of action that RD2 was designed and developed for. Indeed, a significant reduction of Aβ oligomer concentration in fraction 10 of fractionated brain homogenate was found.[21]

To further substantiate the rationale that RD2 will show the same efficacy also in AD patients, we demonstrated that the Aβ oligomers obtained from brains of the APP/PS1 mouse model and from human patients do have very similar size distributions, suggesting that the target Aβ oligomer has similar properties in both sources. The range of Aβ oligomer concentrations found in AD-affected human brain samples overlapped with that of

CellPress
OPEN ACCESS

Table 1. Details of human brain donors

	Gender	Age	Braak	Amyloid	*Post mortem* Interval
AD1	M	77	5	C	4:30 h
AD2	M	91	4		4:15 h
AD3	F	85	6	C	4:00 h
AD4	F	96	5		3:30 h
AD5	F	83	6	C	4:55 h
AD6	M	84	6	C	4:35 h
NC1	M	84	1	O	7:05 h
NC2	M	93	1	O	5:05 h
NC3	F	78	2		7:30 h
NC4	F	78	1	A	7:10 h

the APP/PS1 mouse model. This similarity in Aβ oligomers in the animal experiment and in human patients may already give some degree of confidence to the translation of the pre-clinical efficacy data for clinical trials.

Even more confidence is obtained from the direct observation of RD2-eliminating Aβ oligomers from APP/PS1 mouse brain homogenate *ex vivo* (Figure 3) and from AD patient-derived brain homogenate (Figures 4 and 5). During the *ex vivo* target engagement of RD2 in unfractionated brain homogenates of AD patients, we found a dose- and time-dependent reduction of Aβ aggregates. Two further D-peptides of similar size as RD2 were chosen as controls, D1 and QB37. None of these two peptides showed any dose-dependent effect, indicating that the observed reduction of Aβ oligomer concentration was indeed specific for RD2. A closer look at earlier time points of homogenate at a 1:20 dilution with 20 μM RD2 (Figure 5) revealed that the majority of the reaction took place in the first 40 min of pre-incubation time. Importantly, no reaction was observed without pre-incubation time, which means that no further reaction took place during the incubation with capture antibody on the sFIDA assay plate. It also shows that the observed effect is not attributed to any interaction of RD2 with the assay setup as such. The brain homogenates that we used here present a very complex matrix due to the abundance of different proteins that become released during the homogenization procedure. A considerable portion of RD2 is possibly bound to proteins other than Aβ, while its positive net charge makes it also likely to interact with nucleic acids, glycoproteins, and membrane constituents, such as phospholipids.[40] In order to describe the time and dose dependency of the reduction of Aβ oligomers by RD2 by global kinetic parameters, a concentration of 4 μM of RD2 considered as bound ("threshold dose") was subtracted from the respective total RD2 concentrations. The best fit was achieved with a double exponential decay model, suggesting a fast and a slow reaction. This could be indicative of different types of Aβ aggregates present in the sample that have different susceptibility to RD2. The calculated reaction rate constants, while generally in the same range, were global only within each sample but could potentially be used to compare different compounds. We observed *ex vivo* target engagement of RD2 in pooled DGC fractions 4 to 6 and in a 1:10 dilution of fraction 10 in human samples as well. Unlike fractions 4 to 6 and unfractionated homogenate, fraction 10 showed a notable decrease of Aβ oligomer concentration without pre-incubating with RD2, indicating that the majority of the reaction either happened within less than a minute or during the interval of about 120 min, in which the capture and first washing steps were completed. The latter, however, seems less likely due to the aforementioned observations in unfractionated brain homogenate. On the one hand, this might be due to high susceptibility to RD2 of the particular oligomers in fraction 10. On the other hand, possible matrix effects should be taken into account. While the overall oligomer concentration was almost equally high in a 1:10 dilution of fraction 10 and a 1:20 dilution of homogenate, DGC fractionation generally results in a reduction of complexity and in a dilution of the total protein concentration.

The dose and time dependence of RD2 activity resembles that of an enzyme. This is well in agreement with the proposed mode of action of RD2. RD2 is designed to stabilize Aβ monomers in their native intrinsically disordered conformation. Aβ oligomers, therefore, are destabilized by RD2, which is ultimately disassembling Aβ oligomers into monomers. Complexes of RD2 with Aβ monomers are transient, but of high affinity in the nanomolar K_D range,[17] and may be called fuzzy complexes.[41] The observed dose- and time-dependent Aβ oligomer elimination by RD2 agrees well with the hypothesis that RD2 is acting similar to a chaperone[42] that folds Aβ oligomers back into natively folded Aβ monomers (Figure S6).

CONCLUSION

sFIDA reliably allowed the reproducible measurement of Aβ oligomer concentrations in *post mortem* brain tissues from transgenic AD mouse models and from human AD patients. The combination of sFIDA with particle-size-dependent fractionation of the respective brain tissue homogenates by DGC allowed the quantitative analysis of the particle size distribution of Aβ oligomers. Although the absolute oligomer concentrations varied between individual AD patients and between the different mouse models by about two orders of magnitude, oligomer size distributions were very similar among human AD samples as well as between human AD samples and transgenic mice. The similarity of size distributions of Aβ oligomers in AD mouse models and humans supports a translational value of beneficial effects for cognition observed in the respective animal model, at least for drug candidates that eliminate Aβ oligomers, especially when animal models have been used that express human wild-type Aβ.

Also, animal and human brain tissue can be used to assay for *ex vivo* target engagement of drug candidates designed for direct Aβ oligomer elimination. Our results on *ex vivo* target engagement of RD2 support the findings of earlier *in vitro* and *in vivo* studies demonstrating the Aβ oligomer elimination activity of RD2.[18,21] After *ex vivo* treatment with RD2, the concentrations of Aβ oligomers in brain homogenates, as measured by sFIDA assay, were reduced. Due to the specificity of sFIDA assay for multimeric Aβ assemblies, reduction of sFIDA-obtained oligomer concentrations suggests monomerization of existing, native Aβ oligomers in the samples. The combination of DGC fractionation and sFIDA provides a general and sensitive tool for characterization and identification of different Aβ (co-)aggregates and further

monitoring of *ex vivo* and *in vivo* target engagement. This allows testing of the efficacy of oligomer-eliminating compounds developed for therapy of AD with a fully physiological composition of Aβ aggregates in addition to conventional target engagement studies with pure, synthetic, or recombinant Aβ oligomers. Here, effective target engagement of the compound RD2 with human samples could be a promising indication of its efficacy in human patients. Safety in humans has been demonstrated already.[43] The principle of using *ex vivo*-obtained oligomers to test efficacy of oligomer-eliminating compounds using sFIDA can be translated to other disease-relevant oligomers, such as α-synuclein or tau oligomers.

Limitations of the study

Our analysis of a limited number of human brain samples revealed variability concerning the individual Aβ oligomer concentrations. Yet, we reliably observed *ex vivo* target engagement of RD2 in samples derived from different donors, supporting the mode of action and the general concept of RD2's *ex vivo* target engagement. The reduction of effective peptide concentrations by potential matrix effects in native human brain homogenates is also a point of concern. In future studies, further attempts may be undertaken to reduce this effect, for example, by using isolated DGC-obtained fractions with reduced matrix content.

STAR★METHODS

Detailed methods are provided in the online version of this paper and include the following:

- KEY RESOURCES TABLE
- RESOURCE AVAILABILITY
  - Lead contact
  - Materials availability
  - Data and code availability
- EXPERIMENTAL MODEL AND SUBJECT DETAILS
  - Animal models
  - Human samples
- METHOD DETAILS
  - Preparation of brain homogenates
  - Density gradient centrifugation
  - Incubation of brain homogenates and DGC fractions with D-peptides
  - Surface-based fluorescence intensity distribution analysis (sFIDA) assay
  - Western blot analysis
- QUANTIFICATION AND STATISTICAL ANALYSIS
  - Analysis of image data
  - Calculation of Aβ oligomer concentration
  - Statistical analysis

SUPPLEMENTAL INFORMATION

Supplemental information can be found online at https://doi.org/10.1016/j.xcrm.2022.100630.

ACKNOWLEDGMENTS

D.W. was supported by grants from the Russian Science Foundation (RSF; project no. 20-64-46027) and by the Technology Transfer Fund of the Forschungszentrum Jülich. D.W. was supported by "Portfolio Drug Research" of the "Impuls und Vernetzungs-Fonds der Helmholtzgemeinschaft." Additional funding was received from Part the Cloud: Translational Research Funding for Alzheimer's Disease (PTC) award number: 18PTC-19-605853 from the Alzheimer's Association. We received funding from the European Union Seventh Framework Program (FP7/2007-2013) under grant agreement 602999 (SYMPATH project) and the Federal Ministry of Education and Research within the projects VIP (03V0641), KNDD (01GI1010A), JPND/BIOMARKAPD (01ED1203H), and NEUROALLIANZ (16GW0099). We were also supported by the programs "Biomarkers Across Neurodegenerative Diseases I + II" of the Alzheimer's Association, Alzheimer's Research UK, and the Weston Brain Institute (11084 and BAND-19-614337). We are also grateful for support from The Michael J. Fox Foundation for Parkinson's Research (14977), the ALS Association, and the Packard Center (19-SI-476). We further received funding from the Deutsche Forschungsgemeinschaft (INST 208/616-1 FUGG, INST 208/794-1 FUGG) and the Helmholtz Association (HVF0079). We thank Luana Camargo and Dominik Honold for technical assistance with the collection of mouse brain samples. We thank Dr. Carsten Korth for providing us the IC16 antibody.

AUTHOR CONTRIBUTIONS

Conceptualization, D.W., B.K., C.Z., J.K., and T.B.; methodology, B.K., C.Z., and M.P.; investigation, B.K.; formal analysis, B.K. and C.Z.; visualization, B.K.; writing – original draft, B.K.; writing – review and editing, all authors; funding acquisition, D.W., O.B., and J.K.; resources, J.K., S.S., O.B., and D.W.; and supervision, T.B., J.K., and D.W.

DECLARATION OF INTERESTS

D.W. is a founder and shareholder of the company Priavoid and member of its supervisory board. D.W. is co-inventor of patents related to the compound RD2. C.Z., D.W., and B.K. are inventors of patent WO2019101250A1 (method for quantifying protein aggregates of a protein misfolding disease in a sample). D.W., O.B., and C.Z. are founders and shareholders of attyloid. D.W. is member of attyloid's supervisory board. These had no influence on the interpretation of the data. All other authors declare no competing interests.

Received: December 8, 2021
Revised: February 28, 2022
Accepted: April 15, 2022
Published: May 17, 2022

REFERENCES

1. Prince, M.J., Wimo, A., Guerchet, M., Ali, G.-C., Wu, Y.-T., Prina, M., et al. (2015). World Alzheimer Report 2015: The Global Impact of Dementia: an Analysis of Prevalence, Incidence, Cost and Trends (Alzheimer's Disease International).
2. 2020 Alzheimer's disease facts and figures. Alzheimer's Dementia *16*, 391–460.
3. Sperling, R.A., Aisen, P.S., Beckett, L.A., Bennett, D.A., Craft, S., Fagan, A.M., Iwatsubo, T., Jack, C.R., Jr., Kaye, J., Montine, T.J., et al. (2011). Toward defining the preclinical stages of Alzheimer's disease: recommendations from the National Institute on Aging-Alzheimer's Association workgroups on diagnostic guidelines for Alzheimer's disease. Alzheimer's Dementia *7*, 280–292. https://doi.org/10.1016/j.jalz.2011.03.003.
4. Hadjichrysanthou, C., Evans, S., Bajaj, S., Siakallis, L.C., McRae-McKee, K., de Wolf, F., and Anderson, R.M.; The Alzheimer's Disease Neuroimaging I (2020). The dynamics of biomarkers across the clinical spectrum of Alzheimer's disease. Alzheimer's Res. Ther. *12*, 74.

5. Walsh, D.M., Klyubin, I., Fadeeva, J.V., Cullen, W.K., Anwyl, R., Wolfe, M.S., Rowan, M.J., and Selkoe, D.J. (2002). Naturally secreted oligomers of amyloid β protein potently inhibit hippocampal long-term potentiation in vivo. Nature *416*, 535–539. https://doi.org/10.1038/416535a.

6. Benilova, I., Karran, E., and De Strooper, B. (2012). The toxic Aβ oligomer and Alzheimer's disease: an emperor in need of clothes. Nat. Neurosci. *15*, 349–357. https://doi.org/10.1038/nn.3028.

7. Elfgen, A., Hupert, M., Bochinsky, K., Tusche, M., Gonzalez de San Roman Martin, E., Gering, I., Sacchi, S., Pollegioni, L., Huesgen, P.F., Hartmann, R., et al. (2019). Metabolic resistance of the D-peptide RD2 developed for direct elimination of amyloid-β oligomers. Sci. Rep. *9*, 5715. https://doi.org/10.1038/s41598-019-41993-6.

8. Wiesehan, K., and Willbold, D. (2003). Mirror-image phage display: aiming at the mirror. Chembiochem *4*, 811–815. https://doi.org/10.1002/cbic.200300570.

9. Funke, S.A., and Willbold, D. (2009). Mirror image phage display - a method to generate D-peptide ligands for use in diagnostic or therapeutical applications. Mol. Biosyst. *5*, 783–786. https://doi.org/10.1039/b904138a.

10. van Groen, T., Wiesehan, K., Funke, S.A., Kadish, I., Nagel-Steger, L., and Willbold, D. (2008). Reduction of Alzheimer's disease amyloid plaque load in transgenic mice by D3, a D-enantiomeric peptide identified by mirror image phage display. ChemMedChem *3*, 1848–1852. https://doi.org/10.1002/cmdc.200800273.

11. van Groen, T., Kadish, I., Funke, S.A., Bartnik, D., and Willbold, D. (2013). Treatment with D3 removes amyloid deposits, reduces inflammation, and improves cognition in aged AβPP/PS1 double transgenic mice. J. Alzheimer's Dis. *34*, 609–620. https://doi.org/10.3233/jad-121792.

12. Funke, S.A., van Groen, T., Kadish, I., Bartnik, D., Nagel-Steger, L., Brener, O., Sehl, T., Batra-Safferling, R., Moriscot, C., Schoehn, G., et al. (2010). Oral treatment with the <scp>d</scp>-Enantiomeric peptide D3 improves the pathology and behavior of Alzheimer's disease transgenic mice. ACS Chem. Neurosci. *1*, 639–648. https://doi.org/10.1021/cn100057j.

13. Leithold, L.H.E., Jiang, N., Post, J., Niemietz, N., Schartmann, E., Ziehm, T., Kutzsche, J., Shah, N.J., Breitkreutz, J., Langen, K.-J., et al. (2016). Pharmacokinetic properties of tandem d-peptides designed for treatment of Alzheimer's disease. Eur. J. Pharm. Sci. *89*, 31–38. https://doi.org/10.1016/j.ejps.2016.04.016.

14. Schartmann, E., Schemmert, S., Ziehm, T., Leithold, L.H.E., Jiang, N., Tusche, M., Joni Shah, N., Langen, K.J., Kutzsche, J., Willbold, D., and Willuweit, A. (2018). Comparison of blood-brain barrier penetration efficiencies between linear and cyclic all-d-enantiomeric peptides developed for the treatment of Alzheimer's disease. Eur. J. Pharm. Sci. *114*, 93–102. https://doi.org/10.1016/j.ejps.2017.12.005.

15. Ziehm, T., Buell, A.K., and Willbold, D. (2018). Role of hydrophobicity and charge of amyloid-beta oligomer eliminating <scp>d</scp>-Peptides in the interaction with amyloid-beta monomers. ACS Chem. Neurosci. *9*, 2679–2688. https://doi.org/10.1021/acschemneuro.8b00132.

16. Olubiyi, O.O., Frenzel, D., Bartnik, D., Glück, J.M., Brener, O., Nagel-Steger, L., Funke, S.A., Willbold, D., and Strodel, B. (2014). Amyloid aggregation inhibitory mechanism of arginine-rich D-peptides. Curr. Med. Chem. *21*, 1448–1457. https://doi.org/10.2174/0929867321666131129122247.

17. Zhang, T., Gering, I., Kutzsche, J., Nagel-Steger, L., and Willbold, D. (2019). Toward the mode of action of the clinical stage all-<scp>d</scp>-enantiomeric peptide RD2 on Aβ42 aggregation. ACS Chem. Neurosci. *10*, 4800–4809. https://doi.org/10.1021/acschemneuro.9b00458.

18. van Groen, T., Schemmert, S., Brener, O., Gremer, L., Ziehm, T., Tusche, M., Nagel-Steger, L., Kadish, I., Schartmann, E., Elfgen, A., et al. (2017). The Aβ oligomer eliminating D-enantiomeric peptide RD2 improves cognition without changing plaque pathology. Sci. Rep. *7*, 16275. https://doi.org/10.1038/s41598-017-16565-1.

19. Leithold, L.H.E., Jiang, N., Post, J., Ziehm, T., Schartmann, E., Kutzsche, J., Shah, N.J., Breitkreutz, J., Langen, K.-J., Willuweit, A., and Willbold, D. (2016). Pharmacokinetic properties of a novel d-peptide developed to be therapeutically active against toxic β-amyloid oligomers. Pharm. Res. *33*, 328–336. https://doi.org/10.1007/s11095-015-1791-2.

20. Kutzsche, J., Schemmert, S., Tusche, M., Neddens, J., Rabl, R., Jurgens, D., Brener, O., Willuweit, A., Hutter-Paier, B., and Willbold, D. (2017). Large-scale oral treatment study with the four most promising D3-derivatives for the treatment of Alzheimer's disease. Molecules *22*, 1693. https://doi.org/10.3390/molecules22101693.

21. Schemmert, S., Schartmann, E., Zafiu, C., Kass, B., Hartwig, S., Lehr, S., Bannach, O., Langen, K.J., Shah, N.J., Kutzsche, J., et al. (2019). Aβ oligomer elimination restores cognition in transgenic Alzheimer's mice with full-blown pathology. Mol. Neurobiol. *56*, 2211–2223. https://doi.org/10.1007/s12035-018-1209-3.

22. Birkmann, E., Henke, F., Weinmann, N., Dumpitak, C., Groschup, M., Funke, A., Willbold, D., and Riesner, D. (2007). Counting of single prion particles bound to a capture-antibody surface (surface-FIDA). Vet. Microbiol. *123*, 294–304. https://doi.org/10.1016/j.vetmic.2007.04.001.

23. Wang-Dietrich, L., Funke, S.A., Kuhbach, K., Wang, K., Besmehn, A., Willbold, S., Cinar, Y., Bannach, O., Birkmann, E., and Willbold, D. (2013). The amyloid-β oligomer count in cerebrospinal fluid is a biomarker for Alzheimer's disease. J. Alzheimers Dis. *34*, 985–994. https://doi.org/10.3233/jad-122047.

24. Kravchenko, K., Kulawik, A., Hulsemann, M., Kuhbach, K., Zafiu, C., Herrmann, Y., Linnartz, C., Peters, L., Bujnicki, T., Willbold, J., et al. (2017). Analysis of anticoagulants for blood-based quantitation of amyloid β oligomers in the sFIDA assay. Biol. Chem. *398*, 465–475. https://doi.org/10.1515/hsz-2016-0153.

25. Kulawik, A., Heise, H., Zafiu, C., Willbold, D., and Bannach, O. (2018). Advancements of the sFIDA method for oligomer-based diagnostics of neurodegenerative diseases. FEBS Lett. *592*, 516–534. https://doi.org/10.1002/1873-3468.12983.

26. Hülsemann, M., Zafiu, C., Kuhbach, K., Luhmann, N., Herrmann, Y., Peters, L., Linnartz, C., Willbold, J., Kravchenko, K., Kulawik, A., et al. (2016). Biofunctionalized silica nanoparticles: standards in amyloid-β oligomer-based diagnosis of Alzheimer's disease. J. Alzheimers Dis. *54*, 79–88. https://doi.org/10.3233/jad-160253.

27. Herrmann, Y., Bujnicki, T., Zafiu, C., Kulawik, A., Kuhbach, K., Peters, L., Fabig, J., Willbold, J., Bannach, O., and Willbold, D. (2017). Nanoparticle standards for immuno-based quantitation of α-synuclein oligomers in diagnostics of Parkinson's disease and other synucleinopathies. Clin. Chim. Acta *466*, 152–159. https://doi.org/10.1016/j.cca.2017.01.010.

28. Brener, O., Dunkelmann, T., Gremer, L., van Groen, T., Mirecka, E.A., Kadish, I., Willuweit, A., Kutzsche, J., Jurgens, D., Rudolph, S., et al. (2015). QIAD assay for quantitating a compound's efficacy in elimination of toxic Aβ oligomers. Sci. Rep. *5*, 13222. https://doi.org/10.1038/srep13222.

29. Wiesehan, K., Buder, K., Linke, R.P., Patt, S., Stoldt, M., Unger, E., Schmitt, B., Bucci, E., and Willbold, D. (2003). Selection of D-amino-acid peptides that bind to Alzheimer's disease amyloid peptide $A\beta_{1-42}$ by mirror image phage display. Chembiochem *4*, 748–753. https://doi.org/10.1002/cbic.200300631.

30. Funke, S.A., Bartnik, D., Gluck, J.M., Piorkowska, K., Wiesehan, K., Weber, U., Gulyas, B., Halldin, C., Pfeifer, A., Spenger, C., et al. (2012). Development of a small D-enantiomeric Alzheimer's amyloid-β binding peptide ligand for future in vivo imaging applications. PLoS One *7*, e41457. https://doi.org/10.1371/journal.pone.0041457.

31. Gulyás, B., Spenger, C., Beliczai, Z., Gulya, K., Kása, P., Jahan, M., Jia, Z., Weber, U., Pfeifer, A., Muhs, A., et al. (2012). Distribution and binding of ^{18}F-labeled and ^{125}I-labeled analogues of ACI-80, a prospective molecular imaging biomarker of disease: a whole hemisphere post mortem autoradiography study in human brains obtained from Alzheimer's disease patients. Neurochem. Int. *60*, 153–162. https://doi.org/10.1016/j.neuint.2011.10.010.

32. Jahan, M., Nag, S., Krasikova, R., Weber, U., Muhs, A., Pfeifer, A., Spenger, C., Willbold, D., Gulyás, B., and Halldin, C. (2012). Fluorine-18 labeling of three novel D-peptides by conjugation with N-succinimidyl-4-[^{18}F]

fluorobenzoate and preliminary examination by postmortem whole-hemisphere human brain autoradiography. Nucl. Med. Biol. *39*, 315–323. https://doi.org/10.1016/j.nucmedbio.2011.09.008.

33. Sehlin, D., Englund, H., Simu, B., Karlsson, M., Ingelsson, M., Nikolajeff, F., Lannfelt, L., and Pettersson, F.E. (2012). Large aggregates are the major soluble Aβ species in AD brain fractionated with density gradient ultracentrifugation. PLoS One *7*, e32014. https://doi.org/10.1371/journal.pone.0032014.

34. Mc Donald, J.M., O'Malley, T.T., Liu, W., Mably, A.J., Brinkmalm, G., Portelius, E., Wittbold, W.M., 3rd, Frosch, M.P., and Walsh, D.M. (2015). The aqueous phase of Alzheimer's disease brain contains assemblies built from ∼4 and ∼7 kDa Aβ species. Alzheimers Dementia *11*, 1286–1305. https://doi.org/10.1016/j.jalz.2015.01.005.

35. Upadhaya, A.R., Lungrin, I., Yamaguchi, H., Fandrich, M., and Thal, D.R. (2012). High-molecular weight Aβ oligomers and protofibrils are the predominant Aβ species in the native soluble protein fraction of the AD brain. J. Cell Mol. Med. *16*, 287–295. https://doi.org/10.1111/j.1582-4934.2011.01306.x.

36. Yang, T., Li, S., Xu, H., Walsh, D.M., and Selkoe, D.J. (2017). Large soluble oligomers of amyloid β-protein from Alzheimer brain are far less neuroactive than the smaller oligomers to which they dissociate. J. Neurosci. *37*, 152–163. https://doi.org/10.1523/jneurosci.1698-16.2016.

37. Hong, W., Wang, Z., Liu, W., O'Malley, T.T., Jin, M., Willem, M., Haass, C., Frosch, M.P., and Walsh, D.M. (2018). Diffusible, highly bioactive oligomers represent a critical minority of soluble Aβ in Alzheimer's disease brain. Acta Neuropathol. *136*, 19–40. https://doi.org/10.1007/s00401-018-1846-7.

38. Lana, E., Gellerbring, A., Jung, S., Nordberg, A., Unger Lithner, C., and Darreh-Shori, T. (2019). Homomeric and heteromeric Aβ species exist in human brain and CSF regardless of Alzheimer's disease status and risk genotype. Front. Mol. Neurosci. *12*, 176. https://doi.org/10.3389/fnmol.2019.00176.

39. Thal, D.R., Walter, J., Saido, T.C., and Fändrich, M. (2015). Neuropathology and biochemistry of Aβ and its aggregates in Alzheimer's disease. Acta Neuropathol. *129*, 167–182. https://doi.org/10.1007/s00401-014-1375-y.

40. Meloni, B.P., Mastaglia, F.L., and Knuckey, N.W. (2020). Cationic arginine-rich peptides (CARPs): a novel class of neuroprotective agents with a multimodal mechanism of action. Front. Neurol. *11*, 108. https://doi.org/10.3389/fneur.2020.00108.

41. Borgia, A., Borgia, M.B., Bugge, K., Kissling, V.M., Heidarsson, P.O., Fernandes, C.B., Sottini, A., Soranno, A., Buholzer, K.J., Nettels, D., et al. (2018). Extreme disorder in an ultrahigh-affinity protein complex. Nature *555*, 61–66. https://doi.org/10.1038/nature25762.

42. Arosio, P., Michaels, T.C.T., Linse, S., Månsson, C., Emanuelsson, C., Presto, J., Johansson, J., Vendruscolo, M., Dobson, C.M., and Knowles, T.P.J. (2016). Kinetic analysis reveals the diversity of microscopic mechanisms through which molecular chaperones suppress amyloid formation. Nat. Commun. *7*, 10948. https://doi.org/10.1038/ncomms10948.

43. Kutzsche, J., Jurgens, D., Willuweit, A., Adermann, K., Fuchs, C., Simons, S., Windisch, M., Humpel, M., Rossberg, W., Wolzt, M., and Willbold, D. (2020). Safety and pharmacokinetics of the orally available antiprionic compound PRI-002: a single and multiple ascending dose phase I study. Alzheimers Dement (N Y) *6*, e12001. https://doi.org/10.1002/trc2.12001.

44. Jager, S., Leuchtenberger, S., Martin, A., Czirr, E., Wesselowski, J., Dieckmann, M., Waldron, E., Korth, C., Koo, E.H., Heneka, M., et al. (2009). Î±-secretase mediated conversion of the amyloid precursor protein derived membrane stub C99 to C83 limits AÎ² generation. J. Neurochem. *111*, 1369–1382. https://doi.org/10.1111/j.1471-4159.2009.06420.x.

45. Jankowsky, J.L., Slunt, H.H., Ratovitski, T., Jenkins, N.A., Copeland, N.G., and Borchelt, D.R. (2001). Co-expression of multiple transgenes in mouse CNS: a comparison of strategies. Biomol. Eng. *17*, 157–165. https://doi.org/10.1016/s1389-0344(01)00067-3.

46. Jankowsky, J.L., Fadale, D.J., Anderson, J., Xu, G.M., Gonzales, V., Jenkins, N.A., Copeland, N.G., Lee, M.K., Younkin, L.H., Wagner, S.L., et al. (2004). Mutant presenilins specifically elevate the levels of the 42 residue β-amyloid peptide *in vivo*: evidence for augmentation of a 42-specific γ secretase. Hum. Mol. Genet. *13*, 159–170. https://doi.org/10.1093/hmg/ddh019.

47. Borchelt, D.R., Davis, J., Fischer, M., Lee, M.K., Slunt, H.H., Ratovitsky, T., Regard, J., Copeland, N.G., Jenkins, N.A., Sisodia, S.S., and Price, D.L. (1996). A vector for expressing foreign genes in the brains and hearts of transgenic mice. Genet. Anal. Biomol. Eng. *13*, 159–163. https://doi.org/10.1016/s1050-3862(96)00167-2.

48. Lee, M.K., Borchelt, D.R., Kim, G., Thinakaran, G., Slunt, H.H., Ratovitski, T., Martin, L.J., Kittur, A., Gandy, S., Levey, A.I., et al. (1997). Hyperaccumulation of FAD-linked presenilin 1 variants *in vivo*. Nat. Med. *3*, 756–760. https://doi.org/10.1038/nm0797-756.

49. Garcia-Alloza, M., Robbins, E.M., Zhang-Nunes, S.X., Purcell, S.M., Betensky, R.A., Raju, S., Prada, C., Greenberg, S.M., Bacskai, B.J., and Frosch, M.P. (2006). Characterization of amyloid deposition in the APPswe/PS1dE9 mouse model of Alzheimer disease. Neurobiol. Dis. *24*, 516–524. https://doi.org/10.1016/j.nbd.2006.08.017.

50. Hong, S., Beja-Glasser, V.F., Nfonoyim, B.M., Frouin, A., Li, S., Ramakrishnan, S., Merry, K.M., Shi, Q., Rosenthal, A., Barres, B.A., et al. (2016). Complement and microglia mediate early synapse loss in Alzheimer mouse models. Science *352*, 712–716. https://doi.org/10.1126/science.aad8373.

51. Kamphuis, W., Mamber, C., Moeton, M., Kooijman, L., Sluijs, J.A., Jansen, A.H.P., Verveer, M., de Groot, L.R., Smith, V.D., Rangarajan, S., et al. (2012). GFAP isoforms in adult mouse brain with a focus on neurogenic astrocytes and reactive astrogliosis in mouse models of Alzheimer disease. PLoS One *7*, e42823. https://doi.org/10.1371/journal.pone.0042823.

52. Kilgore, M., Miller, C.A., Fass, D.M., Hennig, K.M., Haggarty, S.J., Sweatt, J.D., and Rumbaugh, G. (2010). Inhibitors of class 1 histone deacetylases reverse contextual memory deficits in a mouse model of Alzheimer's disease. Neuropsychopharmacology *35*, 870–880. https://doi.org/10.1038/npp.2009.197.

53. Lalonde, R., Kim, H.D., Maxwell, J.A., and Fukuchi, K. (2005). Exploratory activity and spatial learning in 12-month-old APP_{695}SWE/co+PS1/ΔE9 mice with amyloid plaques. Neurosci. Lett. *390*, 87–92. https://doi.org/10.1016/j.neulet.2005.08.028.

54. Rupp, N.J., Wegenast-Braun, B.M., Radde, R., Calhoun, M.E., and Jucker, M. (2011). Early onset amyloid lesions lead to severe neuritic abnormalities and local, but not global neuron loss in APPPS1 transgenic mice. Neurobiol. Aging *32*, 2324.e1. https://doi.org/10.1016/j.neurobiolaging.2010.08.014.

55. Minkeviciene, R., Ihalainen, J., Malm, T., Matilainen, O., Keksa-Goldsteine, V., Goldsteins, G., Iivonen, H., Leguit, N., Glennon, J., Koistinaho, J., et al. (2008). Age-related decrease in stimulated glutamate release and vesicular glutamate transporters in APP/PS1 transgenic and wild-type mice. J. Neurochem. *105*, 584–594. https://doi.org/10.1111/j.1471-4159.2007.05147.x.

56. Davis, J., Xu, F., Deane, R., Romanov, G., Previti, M.L., Zeigler, K., Zlokovic, B.V., and Van Nostrand, W.E. (2004). Early-onset and robust cerebral microvascular accumulation of amyloid β-protein in transgenic mice expressing low levels of a vasculotropic Dutch/Iowa mutant form of amyloid β-protein precursor. J. Biol. Chem. *279*, 20296–20306. https://doi.org/10.1074/jbc.m312946200.

57. Levy, E., Carman, M.D., Fernandez-Madrid, I.J., Power, M.D., Lieberburg, I., van Duinen, S.G., Bots, G.T.A.M., Luyendijk, W., and Frangione, B. (1990). Mutation of the Alzheimer's disease amyloid gene in hereditary cerebral hemorrhage, Dutch type. Science *248*, 1124–1126. https://doi.org/10.1126/science.2111584.

58. Grabowski, T.J., Cho, H.S., Vonsattel, J.P.G., Rebeck, G.W., and Greenberg, S.M. (2001). Novel amyloid precursor protein mutation in an Iowa

CellPress
OPEN ACCESS

family with dementia and severe cerebral amyloid angiopathy. Ann. Neurol. *49*, 697–705. https://doi.org/10.1002/ana.1009.

59. Miao, J., Xu, F., Davis, J., Otte-Höller, I., Verbeek, M.M., and Van Nostrand, W.E. (2005). Cerebral microvascular amyloid β protein deposition induces vascular degeneration and neuroinflammation in transgenic mice expressing human vasculotropic mutant amyloid β precursor protein. Am. J. Pathol. *167*, 505–515. https://doi.org/10.1016/s0002-9440(10)62993-8.
60. Moechars, D., Dewachter, I., Lorent, K., Reversé, D., Baekelandt, V., Naidu, A., Tesseur, I., Spittaels, K., Haute, C.V.D., Checler, F., et al. (1999). Early phenotypic changes in transgenic mice that overexpress different mutants of amyloid precursor protein in brain. J. Biol. Chem. *274*, 6483–6492. https://doi.org/10.1074/jbc.274.10.6483.
61. Van Dorpe, J., Smeijers, L., Dewachter, I., Nuyens, D., Spittaels, K., Van Den Haute, C., Mercken, M., Moechars, D., Laenen, I., Kuiperi, C., et al. (2000). Prominent cerebral amyloid angiopathy in transgenic mice overexpressing the london mutant of human APP in neurons. Am. J. Pathol. *157*, 1283–1298. https://doi.org/10.1016/s0002-9440(10)64644-5.
62. Dominguez-del-Toro, E., Rodriguez-Moreno, A., Porras-Garcia, E., Sanchez-Campusano, R., Blanchard, V., Laville, M., Bohme, G.A., Benavides, J., and Delgado-Garcia, J.M. (2004). An in vitro and in vivo study of early deficits in associative learning in transgenic mice that over-express a mutant form of human APP associated with Alzheimer's disease. Eur. J. Neurosci. *20*, 1945–1952. https://doi.org/10.1111/j.1460-9568.2004.03643.x.
63. Rzepecki, P., Nagel-Steger, L., Feuerstein, S., Linne, U., Molt, O., Zadmard, R., Aschermann, K., Wehner, M., Schrader, T., and Riesner, D. (2004). Prevention of Alzheimer's disease-associated Aβ aggregation by rationally designed nonpeptidic β-sheet ligands. J. Biol. Chem. *279*, 47497–47505. https://doi.org/10.1074/jbc.m405914200.
64. Schemmert, S., Schartmann, E., Honold, D., Zafiu, C., Ziehm, T., Langen, K.J., Shah, N.J., Kutzsche, J., Willuweit, A., and Willbold, D. (2019). Deceleration of the neurodegenerative phenotype in pyroglutamate-Aβ accumulating transgenic mice by oral treatment with the Aβ oligomer eliminating compound RD2. Neurobiol. Dis. *124*, 36–45. https://doi.org/10.1016/j.nbd.2018.10.021.
65. van Groen, T., Kadish, I., Wiesehan, K., Funke, S.A., and Willbold, D. (2009). In vitro and in vivo staining characteristics of small, fluorescent, Aβ42-binding D-enantiomeric peptides in transgenic AD mouse models. ChemMedChem *4*, 276–282. https://doi.org/10.1002/cmdc.200800289.

STAR★METHODS

KEY RESOURCES TABLE

REAGENT or RESOURCE	SOURCE	IDENTIFIER
Antibodies		
Anti-beta-Amyloid Purified (SIGNET) Monoclonal Antibody, Unconjugated, Clone 6E10	Covance	Cat# SIG-39320, RRID:AB_662798
IC16	Dr. Carsten Korth, HHU Düsseldorf[44]	N/A
Mouse Anti-Human beta-Amyloid Monoclonal Antibody, Clone NAB 228	Sigma-Aldrich	Cat# A8354, RRID:AB_476770
Goat anti-Mouse IgG (H+L) Secondary Antibody, HRP	Thermo Fisher Scientific	Cat# 31430, RRID:AB_228307
Biological samples		
Human brain samples	Netherlands Brain Bank	https://www.brainbank.nl/
Chemicals, peptides, and recombinant proteins		
OptiPrep^{TM} Density Gradient Medium	Sigma-Aldrich	Cat# D1556
D1	JPT Peptide Technologies	N/A
QB37	peptides&elephants	N/A
RD2	CBL Patras	N/A
Amyloid β-Protein (1-42)	Bachem	Cat# H-1368
Experimental models: Organisms/strains		
Mouse: B6.Cg-Tg(APPswe,PSEN1dE9) 85Dbo/Mmjax (APP$_{swe}$/PS1ΔE9 (APP/PS1))	The Jackson Laboratory	RRID:MMRRC_034832-JAX
Mouse: Tg(Thy1-APPLon)2Vln (APP$_{Lon}$)	Fred van Leuven	RRID:MGI:3717578
Mouse: C57BL/6-Tg(Thy1-APPSwDutIowa) BWevn/Mmjax (APP$_{SwDI}$)	The Jackson Laboratory	RRID:MMRRC_034843-JAX
Mouse: C57BL/6J (wildtype)	The Jackson Laboratory	RRID:IMSR_JAX:000664
Software and algorithms		
sFIDAta	In-house developed software	N/A
ImageJ 1.51k	National Institutes of Health, USA	RRID:SCR_003070
SigmaPlot 11.0	Systat Software, Germany	RRID:SCR_003210
OriginPro 2017G	OriginLab Corp., USA	RRID:SCR_014212

RESOURCE AVAILABILITY

Lead contact

Further information and requests for resources and reagents should be directed to and will be fulfilled by the Lead Contact, Dieter Willbold (d.willbold@fz-juelich.de).

Materials availability

This study did not generate new unique reagents.

Data and code availability

- This study includes no data deposited in external repositories. The datasets generated and/or analyzed during the current study are available from the corresponding author on request.
- This paper does not report original code.
- Any additional information required to reanalyze the data reported in this work paper is available from the Lead contact upon request.

EXPERIMENTAL MODEL AND SUBJECT DETAILS

Animal models

Three different transgenic Alzheimer's disease (AD) mouse models were used:

APP_{swe}/PS1ΔE9 (APP/PS1, RRID:MMRRC_034832-JAX): This well-characterized double-transgenic model was created by Jankowsky et al.[45,46] by co-expressing two previously described transgenes. The mice express chimeric mouse/human APP_{swe} (mouse APP695 with human Aβ domain and "Swedish" mutation K595M/N596L, first created by Borchelt et al.[47]) and human PSEN1 lacking exon 9[48] under control of mouse prion protein promoters. The mutant PSEN1 shifts the ratio of $A\beta_{40}/A\beta_{42}$ in favor of the more amyloidogenic $A\beta_{42}$. The mice start developing plaques at about 4 months,[49] synaptic loss in the hippocampus at 4 months,[50] astrogliosis[51] and deficits in contextual memory starting at six months of age.[52] Spatial memory, as assessed by the Morris water maze (MWM), has been found to be impaired in 12-month-old mice.[53] APP/PS1 mice do not develop tau tangle pathology, and show only moderate neuronal loss in the vicinity of plaques.[54] Successful *in vivo* target engagement of the oligomer eliminating D-peptide RD2 was demonstrated in a study from Schemmert et al.,[21] with RD2-treated mice showing significant cognitive improvement as well as a reduction of Aβ oligomers. The mice were purchased from Jackson and were bred in-house on a C57BL/6J background. In the current study, three male 18 month-old heterozygous APP/PS1 mice on a C57BL/6J background were used, since they show distinctive cognitive deficits in the Morris water maze (MWM) and full-blown plaque pathology at this age.[49,55]

APP_{SwDI} (RRID:MMRRC_034843-JAX): This mouse model was created by Davis et al.[56] by expressing human APP (isoform 770) containing "Swedish" (K670N/M671L), "Dutch" (E693Q), and "Iowa" (D694N) mutations under the control of the mouse Thy1.2 promoter on a C57Bl/6J background. "Dutch" and "Iowa" mutations are located within the Aβ domain, creating a vasculotropic Aβ variant (E22Q/D23N); single "Dutch" or "Iowa" mutations are associated with severe familial cerebral amyloid angiopathy.[57,58] Heterozygous APP_{SwDI} mice accumulate extensive microvascular fibrillar Aβ deposits starting at six months of age, and diffuse Aβ deposits starting at three months of age, covering most of the forebrain by twelve months of age.[56] In addition, they develop gliosis especially in the thalamus and subiculum, increasing over the course of 24 months. Homozygous APP_{SwDI} mice develop pathology faster and to a greater extent, and cognitive deficits were reported using the Barnes maze.[59] The mice were purchased from Jackson and were bred in-house on a C57BL/6J background. In the current study, three female 25.5-month-old homozygous APP_{SwDI} mice were used.

APP_{Lon} (RRID:MGI:3717578): Transgenic mice overexpressing human APP_{695} with the APPV717I ("London") mutation under control of the mouse Thy-1 promotor were first introduced by Moechars et al.[60] In addition to amyloid plaques starting at about 10 months of age, these mice develop cerebral amyloid angiopathy (CAA) bearing similarity to a subset of human AD cases as they age.[61] APP_{Lon} mice show cognitive impairment starting at six months of age, before plaques become evident.[60,62] The mice were originally obtained from Fred van Leuven, and were bred in-house on a C57BL/6J background. For analysis in the current study, two male and two female heterozygous APP_{Lon} mice of more than 24 months of age were used.

Wildtype C57BL/6J mice (RRID:IMSR_JAX:000664) were used as negative controls. All mice were housed under controlled conditions, with a light/dark cycle of 12/12 h, a temperature of 22°C and 54% humidity. Food and water were available *ad libitum*.

Upon reaching a certain age, as described above, mice were sacrificed, brains were removed, and immediately snap-frozen in isopentane. Brain hemispheres were stored at −80°C until further use.

All procedures involving animals were done in accordance with the German Law on the protection of animals (TierSchG). All animal experiments were performed in accordance with the German Law on the protection of animals (TierSchG §§ 7–9) and were approved by a local ethics committee (LANUV, North-Rhine-Westphalia, Germany, reference number: Az: 84-02.04.2014.362 or Az: 84-02.04.2019.A304).

Human samples

Human brain samples were obtained from The Netherlands Brain Bank, Netherlands Institute for Neuroscience, Amsterdam (NBB, open access: www.brainbank.nl), project number 1116. All Material has been collected from donors for or from whom a written informed consent for a brain autopsy and the use of the material and clinical information for research purposes had been obtained by the NBB. The part of the study involving human tissue samples was approved by the ethics committee of the medical faculty of the Heinrich-Heine-University, Düsseldorf (study number: 6215).

Superior parietal lobule samples of six Alzheimer's disease subjects (AD, 77 to 96 years of age at death, Braak staging 4 to 6) and four non-demented control subjects (NC, 78 to 93 years of age at death, Braak stage 1 to 2) were used for analysis. Details, including Braak staging for neurofibrillary tangles (NFT) and amyloid staging as far as provided by the NBB, are listed in Table 1. Two additional NC samples were provided by NBB, but were excluded from further analyses due to extensive amyloid plaque pathology corresponding to stage "B" on a scale from "O" to "C."

METHOD DETAILS

Preparation of brain homogenates

All homogenates were prepared using a Precellys24 homogenizer (Bertin Instruments, Montigny-le-Bretonneux, France) with CK14 lysing kits (Bertin Instruments) for 2 × 30 s, 6000 rpm under constant cooling by Cryolys cooling unit (Bertin Instruments).

All homogenates were centrifuged at 1200 g for 10 min, and the supernatant was used for analysis. The protein concentration of the homogenate supernatants was assessed by BCA assay. Homogenates were stored at −80°C.

Mouse brain samples

One hemisphere of each animal, excluding the cerebellum, was homogenized at a concentration of 10% w/v in Tris buffer (20 mM Tris, 250 mM NaCl, pH 8.3) containing cOmplete EDTA-free protease inhibitor and PhosSTOP phosphatase inhibitor (Roche, Basel, Switzerland).

Human brain samples

Pieces of around 250 mg were homogenized at a concentration of 10% w/v in TBS (24.7 mM Tris, 136 mM NaCl, 2.7 mM KCl, pH 7.4) containing c Omplete EDTA-free protease inhibitor and PhosSTOP phosphatase inhibitor (Roche, Basel, Switzerland).

Density gradient centrifugation

Discontinuous density gradients of 5% to 50% (w/v) iodixanol (OptiPrep, Sigma-Aldrich, St. Louis, MO, USA) buffered to pH 7.4 with 10 mM sodium phosphate buffer (NaP_i), were prepared as described by Brener et al.,[28] Rzepecki et al.:[63] Layers of 260 μL of 50%, 260 μL of 40%, 260 μL of 30%, 780 μL of 20%, 260 μL of 10% and 100 μL of 5% iodixanol were formed in 11 × 34 mm polypropylene open-top ultracentrifuge tubes (Beckman-Coulter, USA). 100 μL of homogenate supernatant, adjusted to the same amount of total protein within each group (mouse or human), was overlaid on top of each gradient. After centrifugation at 259,000 g, 4°C for 3 h using an Optima MAX-XP ultracentrifuge with TLS-55 rotor (both Beckman Coulter, USA), 14 fractions of 140 μL each were collected from top to bottom.

Incubation of brain homogenates and DGC fractions with D-peptides

RD2 was purchased from CBL Patras (Olenia, Greece). RD2 is a rationally designed derivative of the D-peptide D3 - a compound which was originally identified by mirror-image phage display against monomeric $A\beta_{1-42}$.[10] RD2 has first been characterized by Olubiyi, Frenzel (16). As described earlier, RD2 is effective at eliminating Aβ oligomers *in vitro*[18] and has proven its *in vivo* efficiency in different AD mouse models.[20,21,64] The sequence of RD2 is H-ptlhthnrrrrr-NH_2.

D1 was purchased from JPT Peptide Technologies (Berlin, Germany). It was identified by a mirror-image phage display selection targeting $A\beta_{1-42}$ fibrils[29] and has been shown to bind amyloid plaques in human AD brain tissue sections[29] as well as in transgenic APP/PS1 mouse brain samples.[65] The sequence of D1 is H-qshyrhispaqv-NH_2.

QB37 was purchased from peptides&elephants (Henningsdorf, Germany). Its sequence is H-sytldlsgfrgh-NH_2.

All peptide solutions were freshly prepared in 10 mM NaP_i, pH 7.4, from lyophilized powder for each experiment.

DGC derived fraction 10 from APP/PS1 mouse brain homogenate was incubated with an equal volume of RD2 solutions or 10 mM NaP_i, resulting in a dilution factor of 1:2 during incubation. Incubation was performed at 4 °C at 125 rpm. Samples were drawn from the mixtures after the incubation times indicated in Figure 3, and were flash-frozen and were stored at −80°C until analysis.

For *ex vivo* target engagement experiments involving dilutions of 10% human brain homogenates or DGC derived fractions 10 as substrate, the homogenates/fraction 10 were diluted with 10 mM NaP_i, pH 7.4. To avoid further dilution of DGC fractions 4 to 6, they were pooled and were mixed with a volume of RD2 stock solution making up no more than 10% of the total reaction mixture. Incubation was performed at 4°C, 125 rpm. Freezing and thawing was avoided by separately preparing the reaction mixtures at the indicated times before analysis. Even though freeze-thaw cycles were avoided within a single experiment, samples used for *ex vivo* target engagement experiments generally received additional incubation times at 4°C and additional handling, involving e.g. surfaces of pipette tips and Eppendorf tubes, as compared to the samples displayed in Figures 1 and 2. Therefore, total concentrations of recoverable Aβ oligomers were expected to be lower in *ex vivo* target engagement samples.

Surface-based fluorescence intensity distribution analysis (sFIDA) assay

Preparation

sFIDA assays were performed using 384-well glass-bottom microtiter plates (Sensoplate Plus, Greiner Bio-One GmbH, Frickenhausen, Germany). The well bottom was coated with monoclonal antibody Nab228 (RRID:AB_476770, Sigma-Aldrich, St. Louis, MO, USA) using a multi-step protocol for covalent coupling. The principle of the coupling reactions is described in detail by Kulawik et al.[25] First, the glass surface of the plate was functionalized with APTES ((3-aminopropyl)triethoxysilane; Sigma-Aldrich, St. Louis, MO, USA): A 5% (v/v) solution of APTES in anhydrous toluene (Sigma-Aldrich, St. Louis, MO, USA) was placed in a desiccator, the plate was placed above the solution, and incubated in an argon atmosphere for 1 h. After removing the APTES solution, the plate was dried for 2 h under vacuum.

Next, 20 μL of phosphate buffered saline, pH 7.4 (PBS, Sigma-Aldrich, St. Louis, MO, USA) was added to each well, followed by 20 μL of 4 mM succinimidyl carbonate-poly-(ethylene glycol)-carboxymethyl (PEG, MW 3400, Laysan Bio, Arab, USA) in ddH_2O. The plate was incubated for 1 h at room temperature. After washing five times with ddH_2O, the carboxylic acid groups now present on the glass surface were activated by adding a mixture of 200 mM N-(3-dimethylaminopropyl)-N′-ethylcarbodiimide hydrochloride (EDC; Sigma-Aldrich, St. Louis, MO, USA) and 50 mM N-hydroxysuccinimide (NHS; Sigma-Aldrich, St. Louis, MO, USA) in 0.1 M 2-(*N*-morpholino)ethanesulfonic acid (MES; Carl Roth, Karlsruhe, Germany) and incubating for 30 min. After five washes with ddH_2O, 20 μL of 5 μg/mL Nab228 in 0.1 M sodium hydrogen carbonate (Carl Roth, Karlsruhe, Germany) was added to each well and incubated overnight at 4°C. The wells were washed five times each with Tris buffered saline (TBS; SERVA, Heidelberg, Germany) containing 0.05%

Tween20 (AppliChem, Darmstadt, Germany) (TBS-T) and TBS without Tween20. The plate was blocked with 1% bovine serum albumin (BSA; AppliChem, Darmstadt, Germany) in TBS containing 0.03% ProClin 300 (Sigma-Aldrich, St. Louis, MO, USA) for 1 h at room temperature.

Samples and silica nanoparticle standards (SiNaPs, prepared according to Hülsemann et al.[26]) were added after washing the plate five times with TBS-T and TBS and were incubated for 75 min. Depending on the type of sample and the expected concentration of Aβ oligomers, samples were diluted in TBS beforehand to ensure equal distribution and proper separation of aggregates from each other on the well surface for accurate quantitation: Mouse brain homogenate samples of APP_{SwDI}, APP/PS1 and APP_{Lon} were diluted 1:100, their DGC fractions 1:10; wildtype samples were diluted 1:10, their DGC fractions were not diluted. Human brain homogenates were diluted 1:5, human DGC fractions 1:2. These lower dilutions factors were chosen because of the expected lower total concentration of Aβ oligomers and higher variability in human samples in contrast to transgenic mice. Target engagement samples were used at different dilutions, as indicated in the respective experiments. Drying of the wells would cause artifacts; therefore, a volume of 20 μL was present in the wells before addition of 20 μL sample, standard, or antibody, resulting in a total volume of 40 μL during incubation. Due to the aforementioned assay design, an additional dilution factor of 1:2 of the samples' original concentrations was included to yield the final dilution. SiNaPs of 20 nm diameter, coated with about 30 $A\beta_{1-15}$ peptides per particle, were used at final concentrations ranging from 1 fM to 10 pM as calibration standards for Aβ oligomers. Samples and standards were measured in triplicate.

After sample incubation, the plate was washed three times with TBS. Monoclonal detection antibodies Nab228 (RRID:AB_476770, Sigma-Aldrich, St. Louis, MO, USA) and IC16[44] (kindly provided by Prof. Dr. Carsten Korth, Heinrich Heine University Düsseldorf, Düsseldorf, Germany) were labeled with fluorescent dyes CF633 or CF488A (both), respectively. Nab228-CF633 (epitope: $A\beta_{1-11}$) and IC16-CF488A (epitope: $A\beta_{2-8}$) were diluted to a concentration of 0.625 μg/mL each in TBS containing 0.1% BSA and 0.05% Tween20. The solution was centrifuged at 100,000 g for 1 h at 4°C, and 20 μL of the supernatant was added to each well. After 1 h, the plate was washed twice with TBS. 60 μL TBS containing 0.03% ProClin was added to each well to prevent bacterial growth, and the plate was sealed with plastic foil (SealPlate film, Sigma-Aldrich, St. Louis, MO, USA).

Image data acquisition

14-bit grayscale images of the glass surface were acquired with a multi-color laser of a total internal reflection fluorescence (TIRF) microscope (AM TIRF MC, Leica Microsystems, Wetzlar, Germany), using an oil immersion objective (HC PL APO 100x/1.47 OIL CORR TIRF, Leica, Wetzlar, Germany). TIRF penetration depth was set to 200 nm, exposure time was between 400 and 1000 ms with an EM gain of 800 to 1000. 25 positions in each well were imaged in both channels (Ch0: Ex/Em 635/705 nm; Ch1: Ex/Em 488/525 nm) with an image size of 1000 x 1000 pixels, corresponding to an area of 114 × 114 μm per image. In total, approximately 3% of each well's surface area was recorded. A complete set of 25 images of a representative well is presented in Figure S5. Representative TIRF images shown here (except for Figure S5) are cropped to 25% of their original resolution, and their overall brightness is slightly adjusted to ensure better visibility on computer screens or printouts. Analysis was carried out with full-size, raw image files, as described below.

Western blot analysis

For Western blotting, samples were resolved via SDS-PAGE on 12% tris-tricine gels at a constant 45 mA per gel for 1:45 h using the Mini-PROTEAN Tetra Cell (Bio-Rad, Hercules, USA). Proteins were transferred to a PVDF membrane with a pore size of 0.2 μm (Trans-Blot Turbo Mini PVDF Transfer Pack, Bio-Rad, Hercules, USA) at 25 V, 1.3 A for 7 min, using the Trans-Blot Turbo Transfer System (Bio-Rad, Hercules, USA). Membranes were boiled in PBS for 5 min in a microwave oven after transfer. After cooling down to room temperature, the membranes were incubated in fresh PBS for 5 min and in TBS-T (0.1% Tween20) for 5 min. Membranes were blocked with 2% nonfat dried milk powder in TBS-T for 1 h at room temperature. Anti-Aβ antibodies 6E10 (RRID:AB_662798, Covance, Princeton, USA), Nab228 (RRID:AB_476770, Sigma-Aldrich, St. Louis, MO, USA) or IC16 (HHU Düsseldorf, Germany) were used at a concentration of 1 μg/mL in TBS-T over night at 4 °C. Following three 10 min washes with TBS-T, the membranes were incubated with an HRP-coupled goat anti-mouse IgG (RRID:AB_228307, Thermo Fisher Scientific, Waltham, MA, USA), diluted 1:10000 in TBS-T. After washing three times for 10 min with TBS-T, protein bands were visualized with a ChemiDoc MP System (Bio-Rad, Hercules, USA) using ECL Select substrate (GE Healthcare, Boston, USA).

QUANTIFICATION AND STATISTICAL ANALYSIS

Analysis of image data

Images were analyzed using sFIDAta, an in-house developed software. All images containing artifacts such as scratches or images that were out of focus were excluded from analysis. Cutoff values were calculated for each channel, based on the 0.01% most intense pixels of the negative control (TBS). Pixels whose intensity exceeded this background intensity threshold in both channels at the same location (co-localized pixels) were counted to calculate the sFIDA readout. Means and standard deviations of the triplicates were calculated in sFIDAta.

Calculation of Aβ oligomer concentration

The sFIDA readout of the SiNaP standards was used to perform a linear regression analysis. Concentrations were calculated based on this linear regression, reflecting the concentration of oligomer particles of a certain defined size and number of epitopes. Excel 2010 (Microsoft, USA) and OriginPro 9.4 (OriginLab, USA) were used for calculations and graphs.

Statistical analysis

All data are presented as mean ± standard deviation over triplicate wells in single sFIDA measurements (technical replicates), or mean ± standard deviation over the indicated number (N) of biological replicates. In cases where only few different biological samples were analyzed, or variation between samples was high, technical replicates are shown for each sample separately. The limit of detection (LOD) and lower limit of quantification (LLOQ) were defined as the concentration exceeding that of the blank sample by 3 or 10 standard deviations, respectively. Further statistical analyses were carried out in SigmaPlot 11.0 (Systat Software, Germany) and are summed up in Table S1.

The reaction rate constant k_1 was fitted in SigmaPlot 11.0 based on the assumption that RD2 was present in large excess compared to the Aβ oligomer concentration [O], so that the principles of a pseudo-first order reaction apply:

$$[O]_t = [O]_0 \cdot e^{-kt}$$

with

$$k = k_1[RD2]$$

To calculate k_1 directly and globally, a modified formula for a double exponential decay was used:

$$[O]_t = [O]_{fast} * e^{-k_{1,\ fast}*[RD2_{free}]*t} + [O]_{slow} * e^{-k_{1,\ slow}*[RD2_{free}]*t}$$

Taking into account the observation that a certain threshold concentration of RD2 had to be exceeded to show any effect on oligomers in brain homogenate, the assumed free RD2 concentration $[RD2]_{free}$ was used for kinetic fits instead of the original concentrations. $[RD2]_{free}$ was calculated by subtracting 4 μM from each of the used concentrations, yielding effective concentrations of 1 and 16 μM. Fits were calculated using the mean concentrations of three technical replicates.

Cell Reports Medicine, Volume *3*

Supplemental information

Aβ oligomer concentration in mouse and human brain and its drug-induced reduction *ex vivo*

Bettina Kass, Sarah Schemmert, Christian Zafiu, Marlene Pils, Oliver Bannach, Janine Kutzsche, Tuyen Bujnicki, and Dieter Willbold

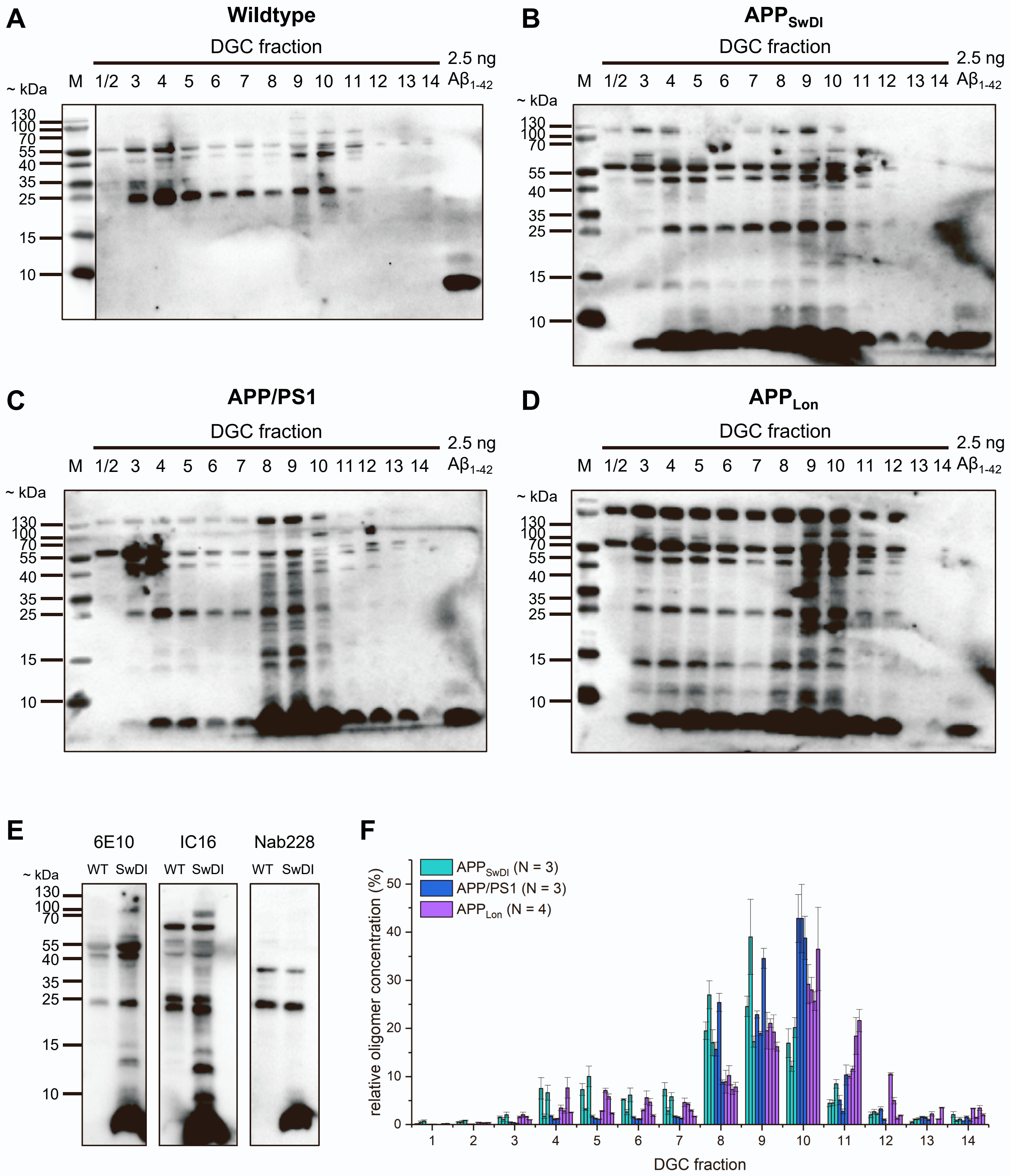

Figure S1: Full Western blots of mouse samples and sFIDA data from all individual animals, related to **Fig 1.**

(A) to (D): Western Blot analysis of undiluted DGC fractions (12 µl per lane) of one representative animal per group in comparison to a synthetic $A\beta_{1\text{-}42}$ standard. Blots were probed with monoclonal antibody 6E10. (A) wildtype, (B) APP_{SwDI} hom., (C) APP/PS1 het., (D) APP_{Lon} het.

(E): Brain homogenates of one wildtype or APP_{SwDI} mouse (30 µg protein per lane) were probed with monoclonal antibodies 6E10, IC16 or Nab228, each at a concentration of 1 µg/ml. (F): Relative Aβ oligomer concentrations of DGC fractions of different mouse models, displaying individual data points for each individual animal.

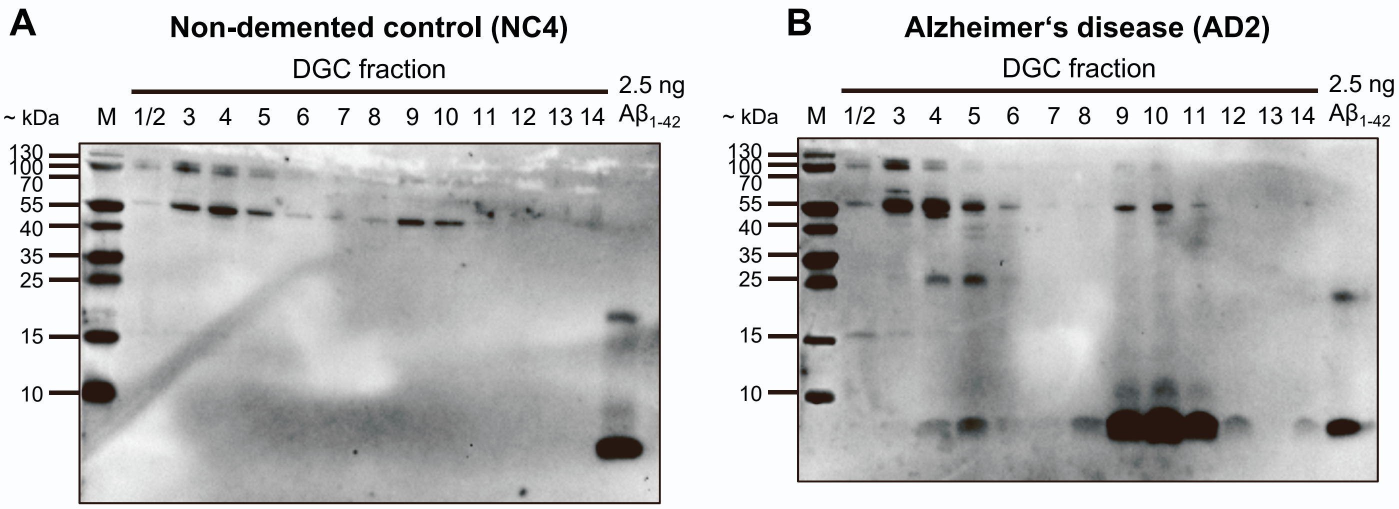

Figure S2: Full Western blots of human samples, related to **Fig 2.**
Western Blot analysis of undiluted DGC fractions (12 µl per lane) of one representative human brain sample per group in comparison to a synthetic $A\beta_{1\text{-}42}$ standard. (A) Non-demented control, (B) Alzheimer's disease. Blots were probed with monoclonal antibody 6E10 at a concentration of 1 µg/ml.

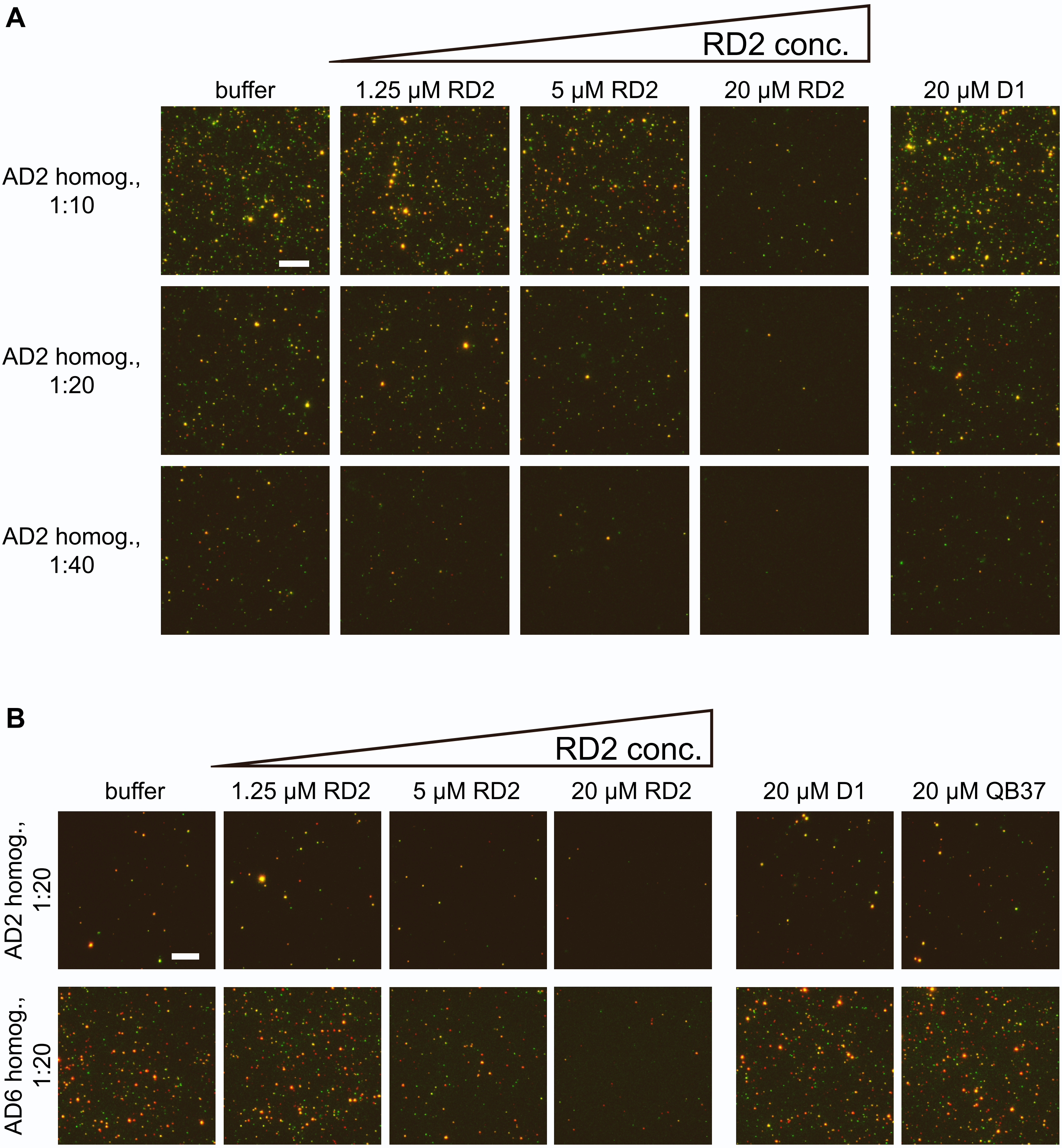

Figure S3: Representative TIRF image sections of human brain homogenate after one day of incubation with D-peptides, related to **Fig 4** and **Fig 5**. Panel (A), corresponding to Fig 4: Homogenates were diluted 1:10, 1:20 or 1:40 during incubation with D-peptides. Final dilution factors during image acquisition were 1:20, 1:40, and 1:80, respectively. Red and green fluorescence channels were merged. The scale bar represents 10 µm. Panel (B): Representative TIRF image sections of human brain homogenate after one day of incubation with D-peptides, corresponding to the data presented in **Fig 5**. Homogenates were diluted 1:20 during incubation with D-peptides. The final dilution factor during image acquisition was 1:40. Red and green fluorescence channels were merged. The scale bar represents 10 µm.

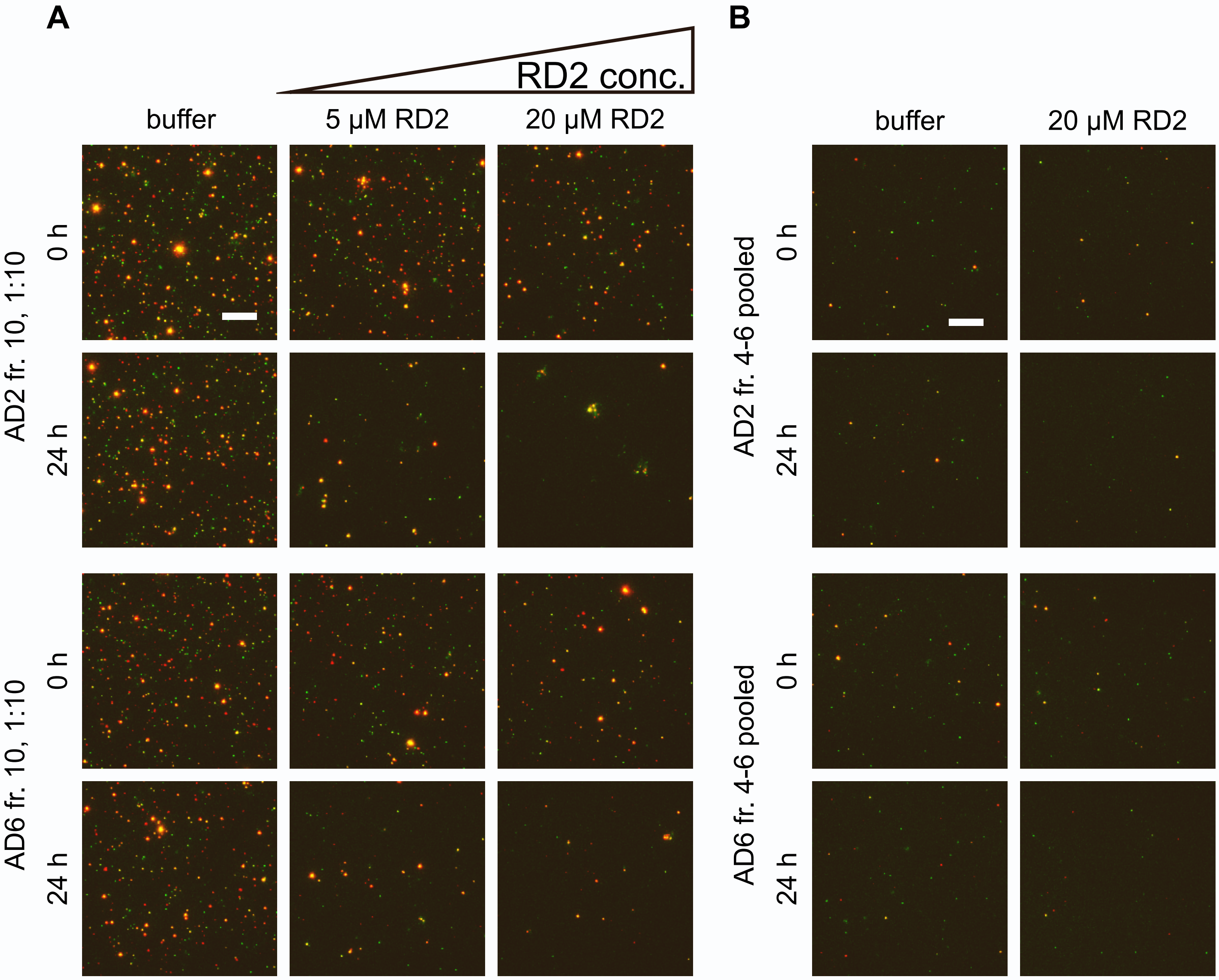

Figure S4, related to **Fig 6:** Representative TIRF image sections of DGC-obtained fractions of human brain homogenate after one day of incubation with RD2. Red and green fluorescence channels were merged. The scale bar represents 10 µm. (A) Images corresponding to the analysis of ex vivo target engagement in fraction 10 of human AD brain homogenates. Fraction 10 was diluted 1:10 during incubation with D-peptides. The final dilution factor during image acquisition was 1:20 (B) Images corresponding to the analysis of ex vivo target engagement in pooled fractions 4 to 6 of human AD brain homogenates. The final dilution factor during image acquisition was 1:2.

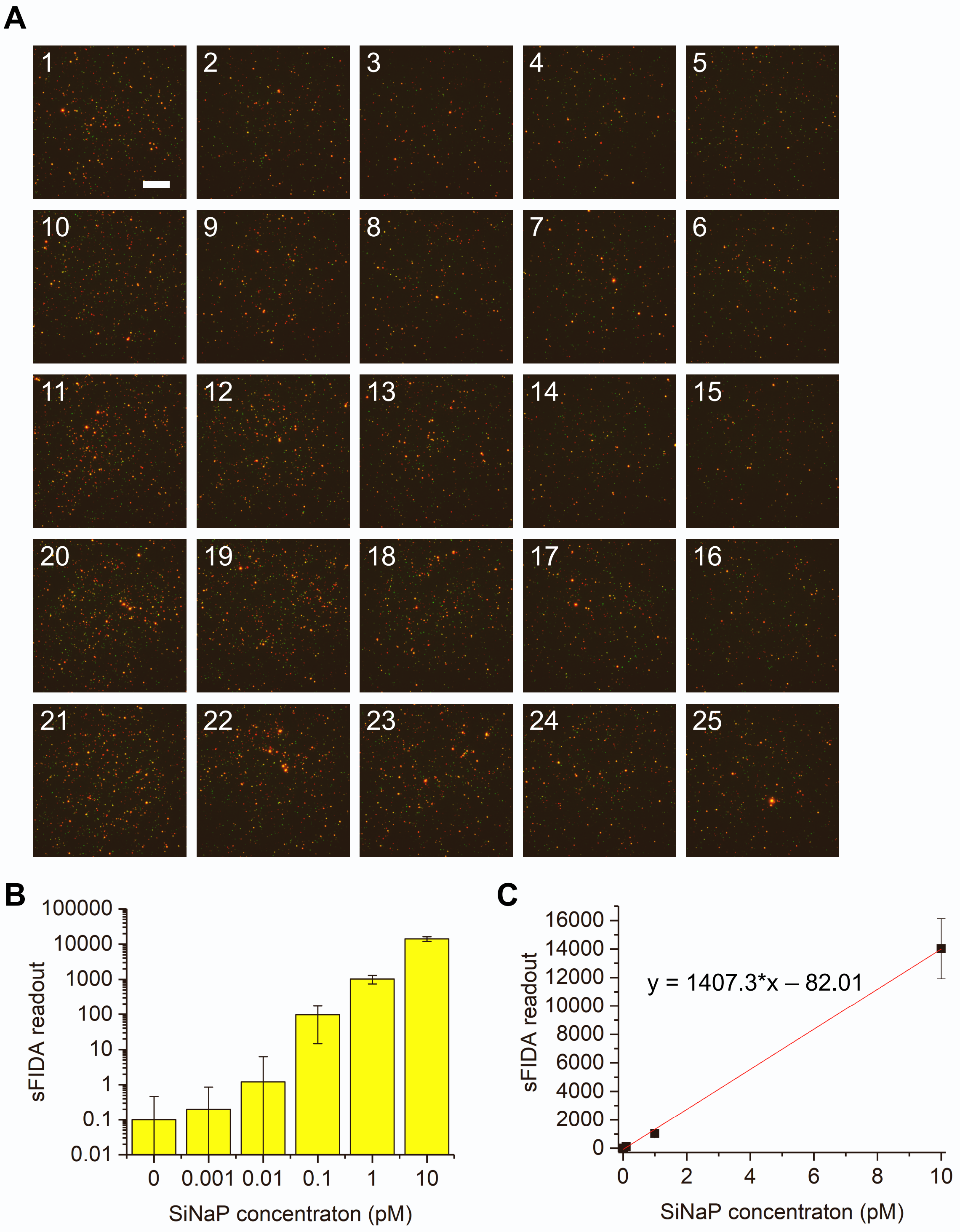

D

$$LOD[fM] = \frac{(sFIDA\,readout_{0\,pM} + 3 \times std\,dev_{0\,pM}) - b}{m} \times 1000$$

$$LLOQ[fM] = \frac{(sFIDA\,readout_{0\,pM} + 10 \times std\,dev_{0\,pM}) - b}{m} \times 1000$$

Figure S5, related to **STAR methods:** Acquisition of sFIDA image data and calculation of concentrations using silica nanoparticle (SiNaP) standards. (A) 25 TIRF images per well are taken in the red and green channel in the order indicated by the numbers 1 to 25. Each sample is measured in triplicate, resulting in a total of 75 images used for analysis. Overlays of red and green channels are shown. The sample depicted here is human AD brain homogenate, diluted 1:20 – corresponding to a final dilution during image acquisition of 1:40. The scale bar represents 20 µm. (B) Co-localized pixels exceeding the background intensity threshold based on the 0.01% pixels with the highest intensity in the buffer control (0 pM SiNaP) are counted to calculate the sFIDA readout. Results in (B) and (C) are displayed as mean ± SD, N = 3 (technical replicates). Please note the logarithmic scale in (B). (C) A serial dilution of SiNaPs coated with a defined number of A$\beta_{1\text{-}15}$ epitopes is used to calculate the concentration of biological samples by linear regression. (D) The limit of detection (LOD) and lower limit of quantitation (LLOQ) are calculated using the slope *m* and Y-axis intercept *b* of the linear regression line.

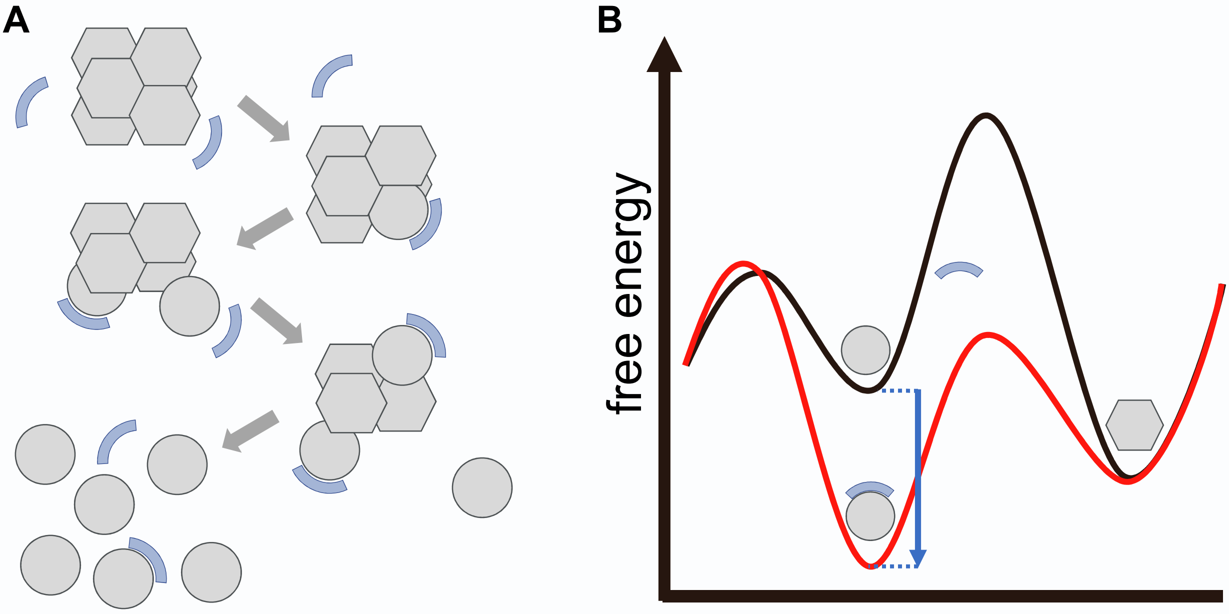

Figure S6, related to **Fig 5:** Proposed mode of action for RD2. (A) RD2 was designed to stabilize Aβ monomers in their native, intrinsically disordered conformation – symbolized by circles. This conformation is distinct from the yet unknown, but certainly highly defined beta-sheet-rich conformation of Aβ building blocks in Aβ oligomers[1] – symbolized by hexagons. Explanation from top to bottom: RD2 molecules – symbolized by circle segments – approach Aβ oligomers. Due to their affinity to Aβ monomers, each RD2 molecule will interact with one of the Aβ building blocks within the Aβ oligomer assembly and thereby pushes its conformation towards the intrinsically disordered monomer conformation. This is incompatible with the oligomer assembly and therefore destabilizing the oligomer assembly. Further destabilization by interaction of additional RD2 molecules with other monomer building blocks, ultimately leads to the complete disassembly of the oligomer into Aβ monomers in their native intrinsically disordered conformation. The affinity between RD2 and Aβ monomers is in the nanomolar KD range although both molecules remain disordered in this transient complex[2], which may therefore be called "fuzzy complex"[3]. The mode of action as shown above, predicts three important properties of RD2. First, because less RD2 molecules are needed than Aβ monomer building blocks are present, RD2 acts at sub-stoichiometric concentrations. We have shown previously, that even at 1:10 sub-stoichiometric ratios, RD2 is efficiently disassembling Aβ oligomers. Second, such destabilization of oligomers by RD2 can be expected to be cooperative. Indeed, we found a Hill coefficient of 3[2]. Third, because RD2 is practically folding Aβ building blocks from oligomers back to their monomeric conformation, RD2 is acting similar to chaperone. Whether this may be called a truly catalytic activity remains to be demonstrated. We called this mode of action "anti-prionic", because it is ultimately disrupting prion-like behaving aggregates[4]. (B) Qualitative and schematic free energy landscape for the anti-prionic mode of action. The black line represents the energy landscape in absence of RD2. Aβ oligomers are more stable than monomers. This allows the formation of oligomers from monomers thermodynamically, although there is a kinetic barrier, which is called primary nucleation and currently under intensive investigation[5,6]. Stabilization of the Aβ monomer by RD2 is lowering the free energy of the Aβ monomer (red line), when in complex with RD2 by the free binding energy (blue arrow) of the complex. Thus, in the presence of RD2, the monomer has a lower free energy as compared to the oligomer, oligomers are disassembled with a reaction rate that is RD2 and Aβ oligomer concentration dependent, exactly as is demonstrated here in this work (Figure 5).

1. König AS, Rösener NS, Gremer L, Tusche M, Flender D, Reinartz E, Hoyer W, Neudecker P, Willbold D, Heise H. Structural details of amyloid β oligomers in complex with human prion protein as revealed by solid-state MAS NMR spectroscopy. J Biol Chem. 2021;296:100499.
2. Zhang T, Gering I, Kutzsche J, Nagel-Steger L, Willbold D. Toward the mode of action of the clinical stage all-D-enantiomeric peptide RD2 on Aβ42 aggregation. ACS Chem Neurosci. 2019;10(12):4800-9.
3. Borgia A, Borgia MB, Bugge K, Kissling VM, Heidarsson PO, Fernandes CB, Sottini A, Soranno A, Buholzer KJ, Nettels D, et al. Extreme disorder in an ultrahigh-affinity protein complex. Nature. 2018;555(7694):61-6.
4. Willbold D, Kutzsche J. Do we need anti-prion compounds to treat Alzheimer's disease? Molecules. 2019;24(12).
5. Noji M, Samejima T, Yamaguchi K, So M, Yuzu K, Chatani E, Akazawa-Ogawa Y, Hagihara Y, Kawata Y, Ikenaka K, et al. Breakdown of supersaturation barrier links protein folding to amyloid formation. Communications Biology. 2021;4(1):120.
6. Meisl G, Kirkegaard JB, Arosio P, Michaels TCT, Vendruscolo M, Dobson CM, Linse S, Knowles TPJ. Molecular mechanisms of protein aggregation from global fitting of kinetic models. Nature Protocols. 2016;11(2):252-72.

Table S1: Summary of statistical analyses, related to **STAR methods, Fig. 3, Fig. 4, Fig. 5, Fig. 6**

Figure	ANOVA	*Post hoc* analysis	Degrees of freedom	
3B	Kruskal-Wallis One-way ANOVA on ranks	Student-Newman-Keuls	4	H: 12.233, P = 0.016
4A	One-way ANOVA	Student-Newman-Keuls	8	F: 7.513, P = <0.001
4B	One-way ANOVA	Student-Newman-Keuls	8	F: 15.667, P = <0.001
4C	One-way ANOVA	Student-Newman-Keuls	8	F: 30.803, P = <0.001
5A	Kruskal-Wallis One-way ANOVA on ranks	Student-Newman-Keuls	5	H: 13.865, P = 0.016
5C	Kruskal-Wallis One-way ANOVA on ranks	Student-Newman-Keuls	5	H: 15.339, P = 0.009
5E	Kruskal-Wallis One-way ANOVA on ranks	Student-Newman-Keuls	5	H: 12.977, P = 0.024
5G	Kruskal-Wallis One-way ANOVA on ranks	Student-Newman-Keuls	5	H: 15.129, P = 0.010
6A	Kruskal-Wallis One-way ANOVA on ranks	Student-Newman-Keuls	2	H: 7.200, P= 0.004
6B	Kruskal-Wallis One-way ANOVA on ranks	Student-Newman-Keuls	2	H: 7.200, P= 0.004
6C	-	t-test, two-tailed	4	t: 16.876, P = <0.001
6D	-	t-test, two-tailed	4	t: 5.880, P = 0.004
